# Viral synergism suppresses *R* gene-mediated resistance by impairing downstream defense mechanisms in soybean

**DOI:** 10.1093/plphys/kiad255

**Published:** 2023-04-26

**Authors:** Mazen Alazem, John Bwalya, Pai Hsuan, Jisuk Yu, Huong Cam Chu, Tessa Burch-Smith, Kook-Hyung Kim

**Affiliations:** Plant Genomics and Breeding Institute, Seoul National University, Seoul 08826, Republic of Korea; The Donald Danforth Plant Science Center, St. Louis, MO 63132, USA; Department of Agricultural Biotechnology, College of Agriculture and Life Sciences, Seoul National University, Seoul 08826, Republic of Korea; The Sainsbury Laboratory, University of East Anglia, Norwich 08826, UK; Plant Genomics and Breeding Institute, Seoul National University, Seoul 08826, Republic of Korea; Department of Agricultural Biotechnology, College of Agriculture and Life Sciences, Seoul National University, Seoul 08826, Republic of Korea; The Donald Danforth Plant Science Center, St. Louis, MO 63132, USA; Plant Genomics and Breeding Institute, Seoul National University, Seoul 08826, Republic of Korea; Department of Agricultural Biotechnology, College of Agriculture and Life Sciences, Seoul National University, Seoul 08826, Republic of Korea; Research Institute of Agriculture and Life Sciences, Seoul National University, Seoul 08826, Republic of Korea

## Abstract

Viral synergism occurs when mixed infection of a susceptible plant by 2 or more viruses leads to increased susceptibility to at least 1 of the viruses. However, the ability of 1 virus to suppress *R* gene-controlled resistance against another virus has never been reported. In soybean (*Glycine max*), extreme resistance (ER) against soybean mosaic virus (SMV), governed by the Rsv3 R-protein, manifests a swift asymptomatic resistance against the avirulent strain SMV-G5H. Still, the mechanism by which Rsv3 confers ER is not fully understood. Here, we show that viral synergism broke this resistance by impairing downstream defense mechanisms triggered by Rsv3 activation. We found that activation of the antiviral RNA-silencing pathway and the proimmune mitogen-activated protein kinase 3 (MAPK3), along with the suppression of the proviral MAPK6, are hallmarks of Rsv3-mediated ER against SMV-G5H. Surprisingly, infection with bean pod mottle virus (BPMV) disrupted this ER, allowing SMV-G5H to accumulate in *Rsv3*-containing plants. BPMV subverted downstream defenses by impairing the RNA-silencing pathway and activating MAPK6. Further, BPMV reduced the accumulation of virus-related siRNAs and increased the virus-activated siRNA that targeted several defense-related nucleotide-binding leucine-rich repeat receptor (NLR) genes through the action of the suppression of RNA-silencing activities encoded in its large and small coat protein subunits. These results illustrate that viral synergism can result from abolishing highly specific *R* gene resistance by impairing active mechanisms downstream of the *R* gene.

## Teaser

Bean pod mottle virus impairs soybean extreme resistance against soybean mosaic virus by interfering with the downstream defense mechanism induced by the *Rsv3 R* gene.

## Introduction

Viral synergism is a key factor in boosting the impact of viral infections, especially with the lack of efficient measures to control viruses. It causes disease outbreaks that result in tremendous losses to agriculture and economies (Nicaise 2014). Viral synergism refers to the status where either or both viruses, typically belonging to different groups, benefit from mixed infections and results in increased viral pathogenicity. The mechanism underlying viral synergy remains largely unexplored on the molecular level. Dominant *R* genes with nucleotide-binding (NB) and leucine-rich repeat (LRR) receptors, termed NLRs, represent a major class of immune receptors that confer durable antiviral resistance in various plants, including soybean (*Glycine max*) (van Wersch et al. 2020; Widyasari et al. 2020). Upon recognition of viral effectors, dominant R proteins activate effector-triggered immunity, leading to a downstream cascade of events that includes the hypersensitive (HR) response, the accumulation of reactive oxygen species (ROS), and the activation of defense hormone signaling pathways (de Ronde et al. 2014; Akhter et al. 2021). In some cases, these responses fail to restrict viral spread beyond the HR lesion, such as in the case of potato virus Y in *Ny-1*-resistant potatoes (*Solanum tuberosum*) (Lukan et al. 2018). In contrast, extreme resistance (ER) prevents viral replication and spread beyond the site of infection due to a swift induction of asymptomatic resistance in which signs such as HR or ROS accumulation are absent (de Ronde et al. 2014; Ross et al. 2021).

*R* genes controlling dominant resistance against soybean mosaic virus (SMV) are divided into 2 groups: *Rsv* genes that confer resistance to the G1 to G7 SMV strains recorded in the United States, and *Rsc* genes that confer resistance to the SC1 to SC22 strains in China. Depending on the strain and the load of the virus, the *R* gene may lead to HR or ER responses (Widyasari et al. 2020; Ross et al. 2021). The soybean cultivar ‘L29’ harbors the NLR *Rsv3* gene, which confers an asymptomatic ER against the SMV-G5H, the avirulent strain of SMV, but is ineffective against the virulent strain G7H (Seo et al. 2014; Tran et al. 2018). Transcriptome profiling revealed that the abscisic acid (ABA) synthesis, RNA-silencing pathway, and callose accumulation are successively induced at the early stage of G5H infection in the Rsv3-mediated ER (Seo et al. 2014; Alazem et al. 2018). However, it is still unclear what defense mechanism governed by Rsv3 leads to full ER. A recent study revealed that the *Rsc4-3* gene in soybean cultivar ‘Dabaima’, a homolog to *Rsv3*, is associated with the cell wall, recognizes the SMV CI effector in the apoplast, and triggers anti-SMV resistance (Yin et al. 2021).

SMV is a positive-sense RNA virus in the *Potyvirus* genus; its ∼10-kb-long genome encodes 1 large polyprotein, which yields 11 proteins after self-cleavage (Hajimorad et al. 2018). The cylindrical inclusion (CI) protein of strain G7H differs by a few amino acids from that of the SMV-G5H CI protein. This difference allows SMV-G7H to avoid Rsv3-recognition and thereby initiate infection of ‘L29’ plants, preventing the induction of defenses against SMV-G7H that are otherwise induced against SMV-G5H (Seo et al. 2009b; Alazem et al. 2018).

The bean pod mottle virus (BPMV) belongs to the genus *Comovirus*, with bipartite positive-sense RNA genomes separately encapsidated in 2 icosahedrally structured virions. The coat proteins (CPs) of *Comovirus*, represented by the type member *Cowpea mosaic virus* (CPMV), are composed of 60 small (CPS) and large (CPL) CP subunits (Di et al. 1999; Mayo and Jones 1999). The BPMV-SMV synergism has been reported for its effects on the performance and behavior of insect vectors (Penaflor et al. 2016). However, the effect of BPMV on soybean resistance with the dominant anti-SMV *R* gene has never been addressed.

Our original goal was to use the BPMV-silencing vector to determine the roles of a few selected soybean genes in Rsv3-mediated ER against SMV-G5H. Instead, we discovered that BPMV offers a novel type of synergism to SMV-G5H by suppressing the highly specific Rsv3 resistance. By interfering with the antiviral RNA-silencing pathway and activating the proviral mitogen-activated protein kinase 6 (MAPK6), BPMV facilitated the replication and cell-to-cell movement of SMV-G5H. Viral suppression of RNA-silencing (VSR) function of BPMV CP subunits, CPL and CPS, is responsible for suppressing Rsv3-mediated ER. These findings underscore a previously unknown facet of viral synergism and identify possible challenges to durable *R* gene-mediated resistance in agricultural settings.

## Results

### BPMV suppresses the specific Rsv3-mediated resistance against SMV-G5H

**Figure 1.**
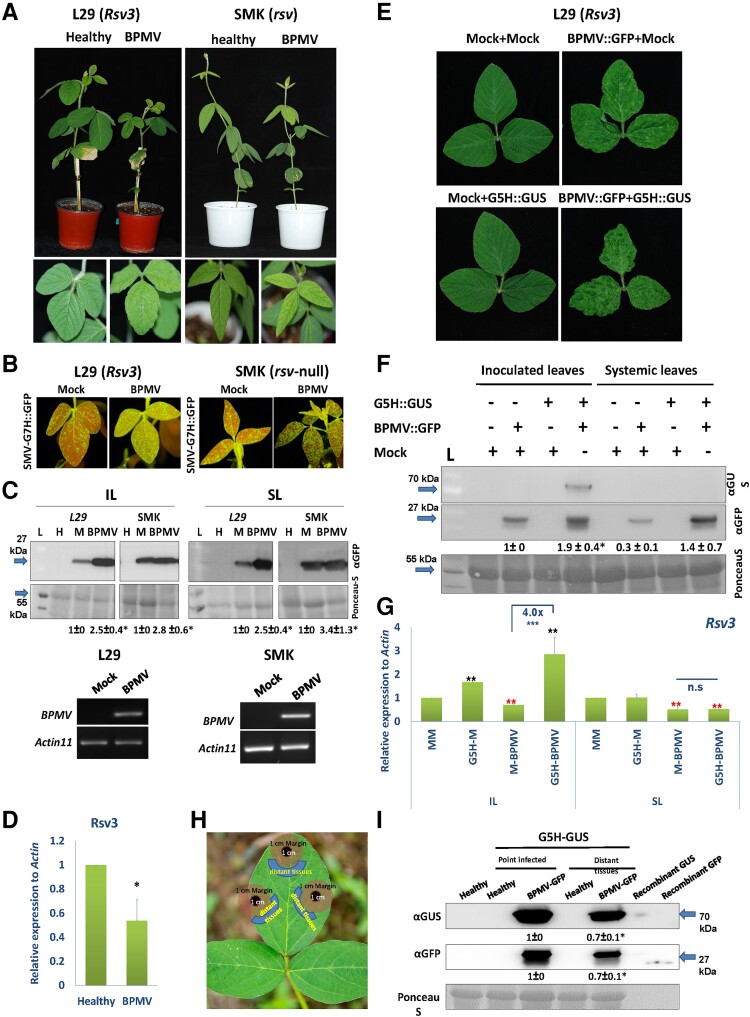
BPMV suppresses Rsv3-mediated ER against SMV-G5H. **A)** Effect of BPMV on the susceptibility of soybean cultivars ‘L29’ (*Rsv3*) and ‘SMK’ (*rsv*) to SMV-G7H infection. Plants were infected with BPMV at the unifoliate leaves, and then at 14 dpi, inoculation with G7H-eGFP was carried out on the 2nd trifoliate leaf. **B)** Accumulation levels of SMV-G7H::eGFP in ‘L29’ plants (left) and ‘SMK’ plants (right) at 10 dpi. **C)** Protein blots of SMV-G7H::eGFP in the inoculated leaves (IL) and the systemically infected leaves (SL), at 5 and 10 dpi, respectively, in mock plants (M) or plants preinfected with BPMV in ‘L29’ plants and ‘SMK’ plants. Ponceau-S was used as a loading control. Lower panels are RT–PCR to detect BPMV movement protein in the systemically infected leaves of ‘L29’ and ‘SMK’ plants (24 dpi: 14 d after BPMV infection plus 10 d after G7H infection). **D)** qPCR relative expression level of *Rsv3* gene in ‘L29’ plants infected with BPMV. Samples were collected from 3 biological replicates, each comprised of 3 plants. *Actin11* was used as an internal control. Values are means + Sd of 3 biological replicates. One-sided Student’s *t* tests were used to determine significant differences at *P* < 0.05 (*). **E)** Symptoms of BPMV::GFP on the systemically infected leaves of ‘L29’ plants with or without G5H::GUS. Unifoliate leaves of 10-d-old plants were infected with BPMV::GFP. After 12 d, the 2nd trifoliate leaf on each plant was infected with G5H::GUS. The systemically infected leaves were photographed 10 d after G5H::GUS infection. **F)** Protein blot for BPMV::GFP and G5H::GUS from ‘L29’ inoculated leaves at 5 dpi and systemically infected leaves at 10 dpi. (−) or (+) indicates the absence or presence of the mock or virus inoculation, respectively. The numbers below the protein blots are the quantification of GFP bands relative to RubScL level, and quantification was carried out by ImageJ. **G)** Relative expression level of *Rsv3* gene in ‘L29’ plants infected with BPMV and/or SMV-G5H from **F)** in the IL or SL. For qPCRs, *Actin11* was used as an internal control. Values are means + Sd of 3 biological replicates. One-sided Student’s *t* tests were used to determine significant differences at *P* < 0.05 (*) and *P* < 0.01 (**). Black and red asterisks indicate significant increase and decrease, respectively. **H)** Experimental design of G5H point inoculation and cell-to-cell movement in ‘L29’ plants fully infected with BPMV. G5H was point inoculated on the 2nd trifoliate leaf (black circles of 1 cm in diameter); margin areas (light orange areas of 1 cm width) separate the inoculated points and the distant regions (blue half circles). Tissues were collected at 5 dpi from black and blue areas to measure G5H accumulation in local and distant tissues. **I)** Protein blots of SMV-G7H::eGFP in point inoculated and the distant regions at 5 dpi. Recombinant GUS and GFP proteins were loaded as positive controls. L denotes the protein ladder, and data represent 1 of 3 biological replicates.

To examine the effect of BPMV on plant defenses against SMV, the ‘L29’ soybean cultivar, which carries the *Rsv3 R* gene, and Somyongkong (‘SMK’) cultivar, an *rsv*-line (Gunduz et al. 2001; Kim et al. 2003), were infected with BPMV. At 14 d postinfection (dpi), BPMV-infected ‘L29’ plants were stunted compared to the healthy plants and had developed only 3 trifoliate leaves with mild mottling symptoms. In contrast, the healthy plants had developed 4 trifoliate leaves (Fig. 1A, left images). The effect of BPMV was weaker on ‘SMK’ plants than on ‘L29’ plants, where ‘SMK’ plants were slightly stunted but also had mild mottling on their upper trifoliate leaves (Fig. 1A, right images). We next inoculated the second trifoliate leaf of each plant with the G7H virulent strain of SMV that expresses enhanced green fluorescent protein (eGFP) (G7H::eGFP; Supplemental Fig. S1A). G7H-eGFP produced stronger GFP fluorescence on the systemically infected leaves in the BPMV-infected ‘L29’ or ‘SMK’ plants compared to the healthy plants (Fig. 1B). Notably, the level of G7H::eGFP was substantially higher in both inoculated and systemically infected leaves of BPMV-infected plants than in mock-treated leaves (Fig. 1C). When BPMV-infected plants were incubated until 24 dpi, time was extended to 24 d and severe stunting and leaf malformations were observed on ‘L29’ plants (Supplemental Fig. S1B).

Interestingly, BPMV infection significantly reduced the expression of *Rsv3* by ∼2-fold (Fig. 1D), which prompted us to test whether BPMV infection interferes with the Rsv3-mediated ER against SMV-G5H in the resistant cultivar ‘L29’. Plants were infected with a BPMV clone expressing GFP; at 12 dpi, the second trifoliate leaves were infected with SMV-G5H expressing GUS (SMV-G5H::GUS; Supplemental Fig. S1, A and C). Although ‘L29’ leaves infected with SMV-G5H::GUS remained symptomless, those infected with BPMV::GFP developed strong mottling symptoms that were enhanced in the mixed infection of BPMV::GFP and SMV-G5H::GUS (Fig. 1E). Surprisingly, SMV-G5H::GUS accumulated in the inoculated leaves but not in the systemically infected leaves (Fig. 1F). In addition, in the inoculated and systemically infected leaves, BPMV::GFP accumulation was enhanced in the mixed infection with SMV-G5H::GUS (Fig. 1F). Interestingly, *Rsv3* expression was increased in the inoculated leaves of BPMV/G5H mixed infection by ∼4-fold compared with the BPMV-infected plants but remained unchanged in the systemic leaves of mixed-infected plants (Fig. 1G). This implies that the accumulation of SMV-G5H in ‘L29’ led to continuous induction of *Rsv3* expression. These findings indicate that BPMV infection interfered with the anti-G5H defense mechanisms in soybean cultivar ‘L29’ that led to the suppression of Rsv3 resistance against the G5H avirulent strain in inoculated leave. We next examined whether BPMV facilitates cell-to-cell movement of G5H in the Rsv3-containing plants. BPMV fully-infected plants were point-infected with G5H in areas less than 1 cm in diameter. At 5 dpi, adjacent areas that are >1 cm away from the point of infection were examined for G5H accumulation (Fig. 1H). Protein blots showed that BPMV broke the Rsv3-local resistance facilitating G5H movement outside the infection zone (Fig. 1I).

To investigate which defense pathway is impaired by BPMV infection, the SA, ABA, and RNA-silencing antiviral defenses were examined upon BPMV infection in both ‘L29’ and ‘SMK’ plants. In ‘L29’ plants, the SA-related genes Enhanced Disease Susceptibility1 (*EDS1*), Isochorismate Synthase1 (*ICS1*), Nonexpresser of Pathogenesis-Related genes 1 (*NPR1*), and Phytoalexin Deficient4 (*PAD4*), as well as the RNA-silencing genes; Dicer-Like (*DCLs*), RNA-Dependent RNA Polymerase (*RDRs*), and most of the Argonaute (*AGO*) genes were strongly upregulated by several folds compared with healthy plants where expression of some genes was upregulated by ∼20 to 60-folds (Supplemental Fig. S2, A, C, and E). A mild upregulation was observed for several genes in both pathways in ‘SMK’ plants (Supplemental Fig. S2, B, D, and F). Notably, the expression of a small number of *AGO* genes was reduced by BPMV in ‘SMK’ plants (Supplemental Fig. S2F). In ‘L29’ plants, the expression of most ABA-related genes, which are critical for Rsv3-mediated ER against SMV-G5H, was slightly greater in BPMV-infected plants than in healthy ones (Supplemental Fig. S2G). This upregulation was only significant for *ABA1_5700* and *AAO3_5100* in ‘SMK’ plants (Supplemental Fig. S2H). These findings suggest that although plants responded to BPMV with strong transcriptional activation of the SA and the RNA-silencing pathways, BPMV was able to suppress such defenses.

### BPMV large and small CP subunits have VSR activity

**Figure 2.**
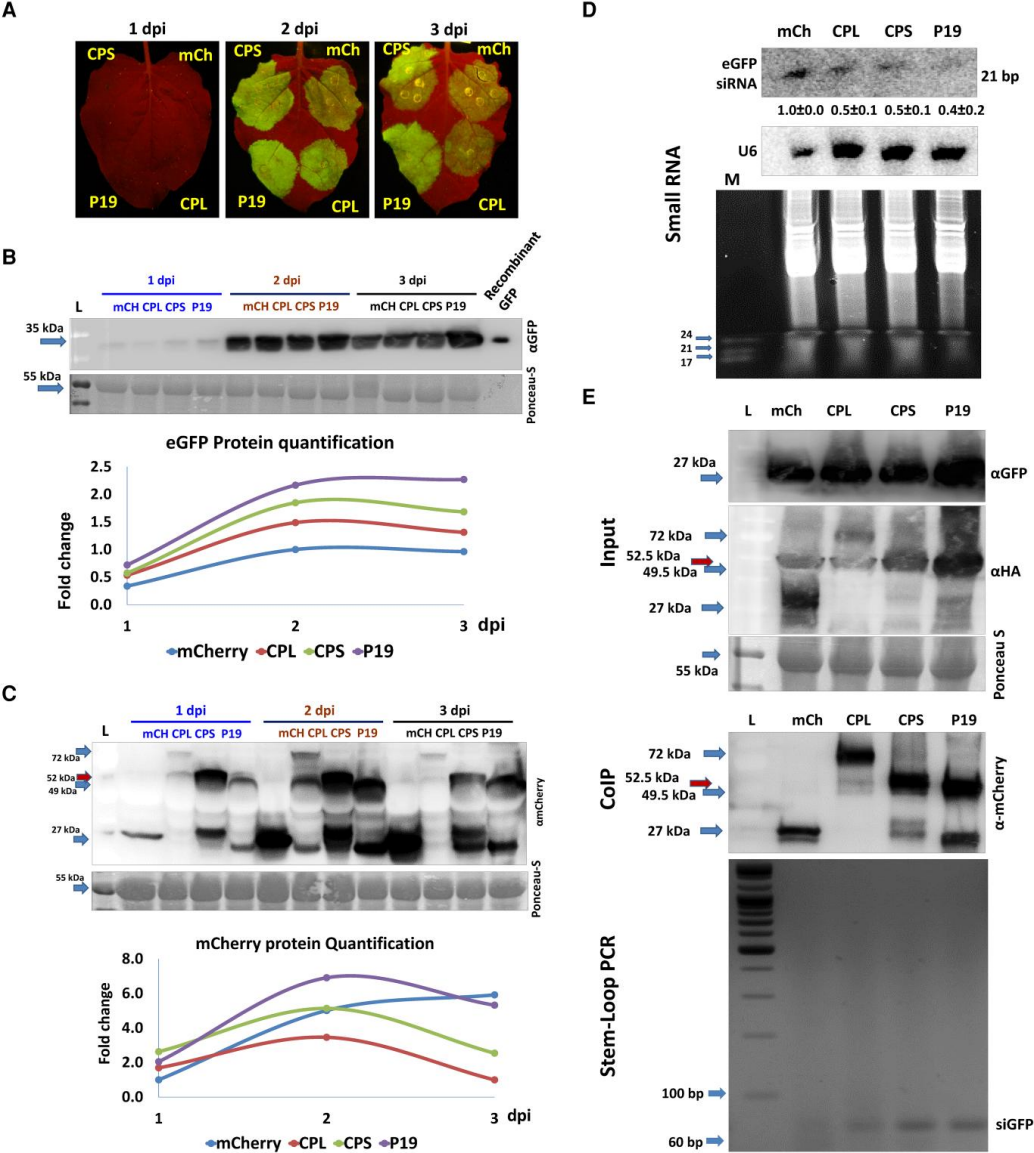
Viral suppressor of RNA-silencing activity of BPMV coat protein subunits CPL and CPS in *N. benthamiana* plants. **A)** GFP fluorescence levels in *N. benthamiana* transiently expressing CPL, CPS, P19, or mCherry (mCh) with eGFP at 1, 2, and 3 dpi. **B)** Protein blot of *N. benthamiana* from **A)** with GFP antibody; free GFP recombinant protein was loaded as a positive control. **C)** Protein blot from **A)** for mCherry (27 kDa), CPL (72 kDa), CPS (51.5 kDa), and P19 (49.5 kDa) at 1, 2, and 3 dpi. Blot represents 1 of 3 biological replicates, L denotes the protein ladder, and Ponceau-S was used as the loading control. The charts below **B)** and **C)** are the quantifications of the bands, which were carried out by ImageJ using RubScL as an internal control. **D)** Northern blot of eGFP siRNA from *N. benthamiana* leaves at 2 dpi; upper panel is eGFP siRNA of 21 bp size and U6 was used as a loading control. The lower panel is the total small RNA, and M is the miRNA marker showing 17-, 21-, and 24-mer bands. **E)** Stem–loop RT–PCR for a siRNA eGFP coimmunoprecipitated from a mix of eGFP with CPL, CPS, or P19 proteins from **A)**, and the input panel represents GFP and HA protein blots for samples collected at 2 dpi from the leaves shown in **A)**. The CoIP panel is the protein blot (upper) for the coimmunoprecipitated mCherry, CPL, CPS, or P19 proteins by anti-HA antibody and the stem–loop RT–PCR (lower) for a siGFP from RNA samples isolated from the coimmunoprecipitated proteins. L denotes the protein ladder. Each protein or RNA blot represents 3 biological replicates.

A possibility of posttranscriptional suppression of antiviral defenses is the interference with the RNA-silencing pathway by BPMV-encoded VSRs. The CPS of CPMV, a member of the same genus, has VSR activity in *Nicotiana benthamiana* (Liu et al. 2004). To determine the VSR activity of viral proteins, their ability to enhance the transient expression of marker proteins such as GFP should be examined. To investigate that BPMV's, CPL, or CPS carrying a C-terminal HA-mCherry protein tag were transiently co-expressed with pBin-eGFP in *N. benthamiana* leaves. The potent VSR P19 from the tomato bushy stunt virus was used as a positive control. At 2 dpi, both CPL and CPS showed VSR activity compared with the control (HA-mCherry with eGFP), whereas the VSR activity of P19 extended to 3 dpi (Fig. 2A). This observation was confirmed by a protein blot where greater eGFP accumulated in the presence of CPL and CPS than in the controls (Fig. 2B). In this transient assay, CPL and CPS were less stable than mCherry or P19 as their accumulation levels declined at 3 dpi (Fig. 2C). CPL and CPS exert VSR activity that relies on protein stability in the transient assay.

Northern blot showed that these VSRs reduced eGFP-derived small interfering RNAs (siRNAs) and total sRNA compared with the mCherry control (Fig. 2D). To determine if CPL or CPS has any siRNA sequestering function similar to that reported for P19 (Li and Wang 2019), co-immunoprecipitation was conducted, followed by sRNA extraction from the precipitated VSRs. Stem–loop RT–PCR targeted specific siRNA from eGFP and showed that all VSRs have siRNA sequestering ability (Fig. 2E).

### VSRs suppress the Rsv3-mediated ER by impairing the RNA-silencing pathway

To determine whether CPL or CPS increases SMV-G5H virulence, the 2 genes and P19 were cloned downstream of eGFP in the pSMV-G5H::eGFP infectious clone (Supplemental Fig. S1C). At 18 dpi, the susceptible soybean cultivar ‘Lee74’ showed no noticeable difference in the growth of plants infected with SMV-G5H::eGFP, SMV-G5H::eGFP::CPS, or SMV-G5H::eGFP::CPL, but plants infected with SMV-G5H::eGFP::P19 were stunted (Supplemental Fig. S3A). eGFP fluorescence was high in the leaves systemically infected with the 4 virus variants (Supplemental Fig. S3B). A protein blot showed that these VSRs increased the accumulation of SMV-G5H compared with the control SMV-G5H::eGFP (Supplemental Fig. S3C). We inferred that, similar to P19 but with a lesser strength, CPL and CPS impaired the RNA-silencing pathway and therefore caused the increased accumulation of SMV-G5H in the susceptible soybean cultivar ‘Lee74’.

**Figure 3.**
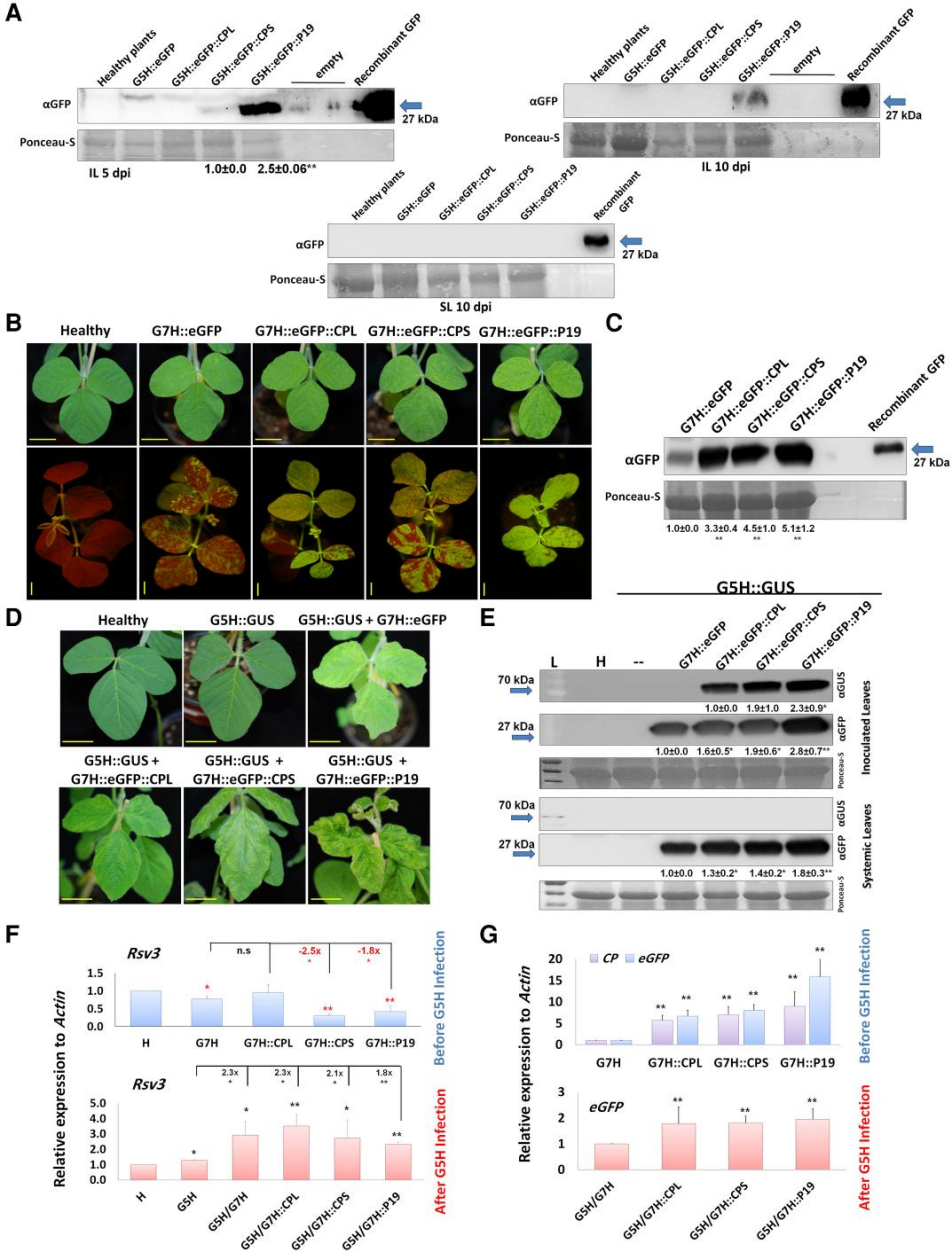
CPL and CPS subunits of BPMV are responsible for impairing Rsv3-mediated ER against SMV-G5H. **A)** Protein blots for SMV-G5H::eGFP constructs expressing CPL, CPS, or P19 from ‘L29’ plants in the inoculated leaves (IL) at 5 and 10 dpi (left and right panels, respectively) and the systemically infected leaves (SL) at 10 dpi (lower panel). GFP recombinant protein was loaded as a positive control. The blot is representative of 3 biological replicates. **B)** Visual symptoms and GFP fluorescence in the ‘L29’ leaves systemically infected with SMV-G7H::eGFP expressing CPL, CPS, or P19. The 2nd unifoliate leaves were photographed at 12 dpi. Scale bar = 2 cm. **C)** Protein blot of eGFP from protein samples extracted from ‘L29’ leaves systemically infected with SMV-G7H::eGFP expressing CPL, CPS, or P19 at 12 dpi. **D)** Symptoms developed on ‘L29’ upper leaves [from **A)**] infected with SMV-G5H::GUS 10 dpi. Scale bar = 2 cm. **E)** Protein blot of eGFP (for SMV-G7H::eGFP::VSRs) and GUS (for SMV-G5H::GUS) from the SMV-G5H::GUS–inoculated and SMV-G5H::GUS–systemically infected leaves, at 10 dpi. L denotes the protein ladder, Ponceau-S is the loading control, and blots are representative of 3 biological replicates. The numbers below the panels are the quantification of the bands relative to the RubScL level revealed by Ponceau-S, and quantification was carried out by ImageJ. **F)** Relative expression level of *Rsv3* gene before and after SMV-G5H::GUS infection in ‘L29’ plants infected with SMV-G7H::eGFP::VSRs. **G)** Relative expression of SMV-G7H–encoded genes eGFP and CI in ‘L29’ plants infected with the SMV-G7H::eGFP::VSR alone (top chart) or after SMV-G5H::GUS infection (bottom chart). *Actin* was used as an internal control. Values are means + Sd of 3 biological replicates. Statistical analysis was carried out as described in the legend of Fig. 1; * and ** indicate a significant difference at *P* < 0.05 and 0.01, respectively.

Next, we inoculated the resistant ‘L29’ plants with sap extracted from infected ‘Lee74’ plants. CPS, and to a greater degree P19, allowed SMV-G5H::eGFP to accumulate in the inoculated leaves of ‘L29’ at 5 dpi (Fig. 3A, left panel). When the infection time was increased to 10 d, only SMV-G5H::eGFP::P19 could be detected but a level lower than that at 5 dpi (Fig. 3A, right panel). No (SMV) eGFP was detected in the upper, uninoculated leaves of any of the plants (Fig. 3A, lower panel), suggesting that impairment of RNA silencing by P19, CPS, or CPL suppresses the local resistance mediated by Rsv3 against SMV but does not result in systemic infection by the virus in ‘L29’ plants. In addition, ‘L29’ plants were able to recover over time which suggests that Rsv3-mediated resistance was able to overcome the infection of G5H::eGFP::VSRs.

### Constitutive expression of CPL and CPS suppresses local Rsv3-mediated ER

We hypothesized that the constitutive expression of CPL, CPS, or P19 might completely suppress the Rsv3-mediated resistance against G5H. To challenge this hypothesis, we aimed to infect ‘L29’ plants with the virulent G7H strain expressing CPL, CPS, and P19 that guarantees a constitutive expression of these VSRs in the entire plant. Similar to their effect on SMV-G5H virulence, CPL, CPS, and P19 allowed SMV-G7H::eGFP to fully infect ‘Lee74’ plants with P19 expression leading to the most severe stunting (Supplemental Fig. S4A). CPL, CPS, and P19 allowed G7H to accumulate more eGFP than the SMV-G7H::eGFP control in the systemically infected leaves (Supplemental Fig. S4, B and C).

‘L29’ plants were next infected with infectious sap containing SMV-G7H::eGFP::CPL, SMV-G7H::eGFP::CPS, or SMV-G7H::eGFP::P19. At 12 dpi, mosaic symptoms and GFP signal were observed, and their severity was highest with SMV-G7H::eGFP::P19 followed by SMV-G7H::eGFP::CPS, and SMV-G7H::eGFP::CPL (Fig. 3B). This observation was confirmed by protein blots, where the accumulation of eGFP was associated with the observed fluorescence signal (Fig. 3C). Once ‘L29’ was completely infected with SMV-G7H clones, the second trifoliate leaf of each plant was sap-infected with SMV-G5H::GUS. At 10 dpi, the mosaic symptoms were strong in the systemically infected leaves (the 4th trifoliate leaves), with P19 infection causing abnormalities in the newly emerging leaves and with SMV-G5H::GUS-inoculated leaves appearing healthy (Fig. 3D). SMV-G5H::GUS accumulated locally to high levels in the inoculated leaves of plants infected with SMV-G7H::eGFP::VSRs at 10 dpi. However, SMV-G5H::GUS was undetectable in any of the upper leaves that were systemically infected with G7H::eGFP::VSRs (Fig. 3E). This indicates that interfering with the RNA-silencing pathway impairs only the local resistance mediated by Rsv3.

Interestingly, the expression of *Rsv3* was significantly reduced by infection with the SMV-G7H constructs, except for G7H::eGFP::CPL. Following SMV-G5H::GUS infection, however, *Rsv3* was increased considerably in the mix infection of SMV-G7H variants and SMV-G5H::GUS (Fig. 3F). Accumulation of *eGFP* or *CP* transcripts encoded in G7H was comparable among plants infected with SMV-G7H::VSR chimeras with ∼6-fold higher than in G7H alone. This increase, however, was reduced to 2-fold with SMV-G5H::GUS infection (Fig. 3G). Thus, the VSRs suppress the Rsv3-mediated ER against G5H only in the inoculated leaves.

### Knocking down RNA-silencing genes increases the susceptibility of Rsv3-plants to SMV-G5H

The ability of BPMV VSR proteins to suppress the *Rsv3*-mediated resistance prompted us to test whether their effect is related to RNA silencing. We, therefore, used BPMV to silence *RDR1a* and *RDR6a* in Rsv3-plants, then challenged them with G5H-GUS. The empty vector (EV) of BPMV significantly increased the expression of *RDR1a* and *RDR6a* by more than 45-fold compared with healthy plants. Silencing either gene, however, significantly reduced their expression by several folds compared to BPMV-EV plants (Supplemental Fig. S5A). G5H-GUS accumulated more in *RDR1a*- and *RDR6a*-silenced plants than in the BPMV-EV plants. It was, however, unable to accumulate in healthy plants (Supplemental Fig. S5B). This result supports that the RNA-silencing pathway is critical in Rsv3-mediated resistance.

### Impacts of SMV::VSRs infection on small RNA biogenesis in soybean

To analyze sRNA profiles, sRNA pools were purified from healthy, SMV-G7H::VSRs infected plants and were subsequently analyzed via sRNA sequencing. After quality trimming and size filtering, an average of 20 million clean reads of 18 to 30 nt sRNA were recorded for the libraries with a clean-read percentage of ∼81% (Supplemental Data Set S1). Infection with SMV-G7H::eGFP or its variants slightly reduced the total sRNA reads compared to that in healthy plants (Supplemental Fig. S6A). Interestingly, reduction in virus-derived siRNA (vsiRNA) levels was enhanced by G7H::VSRs compared with G7H::eGFP (Supplemental Fig. S6B).

**Figure 4.**
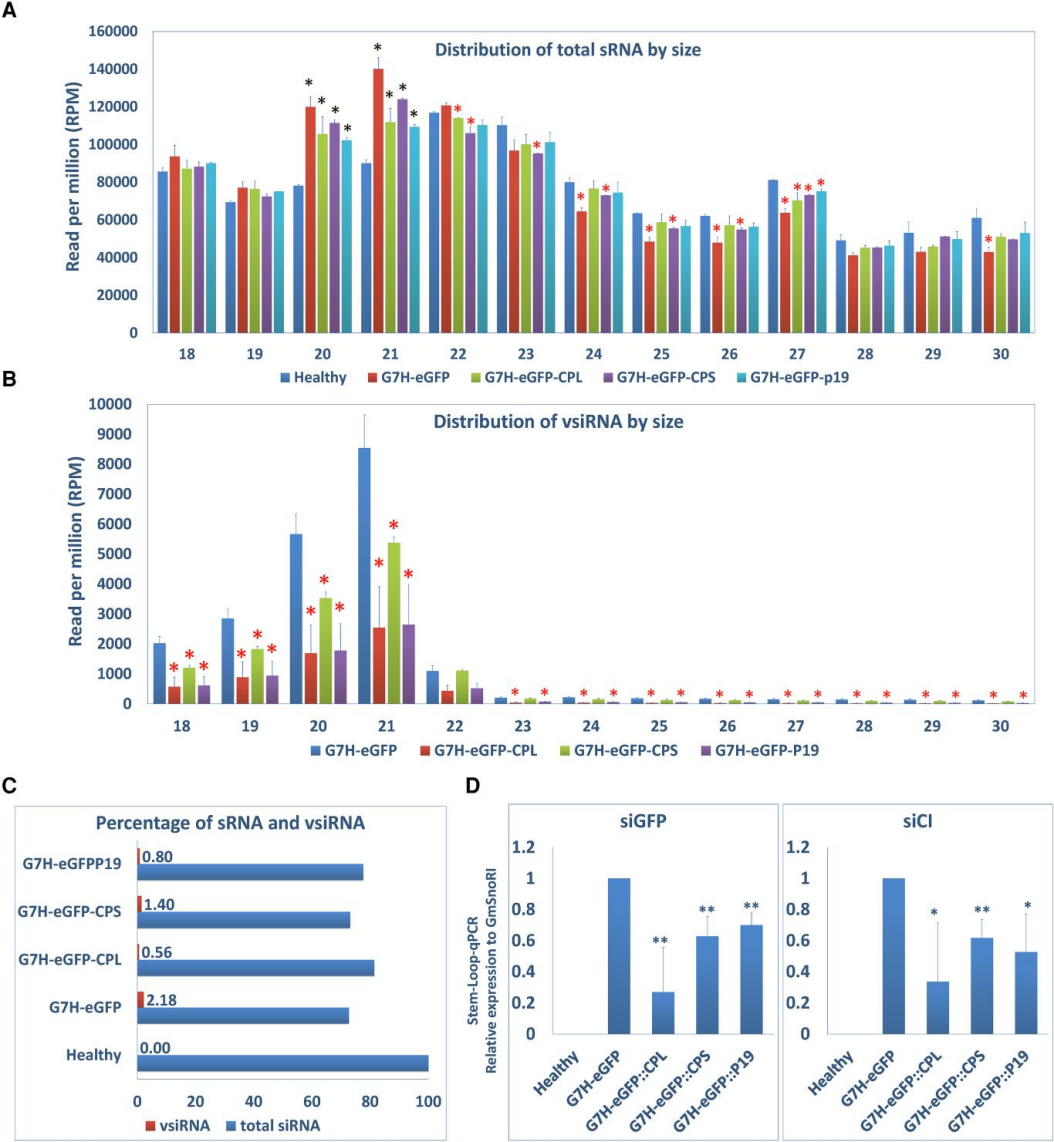
Characteristics of sRNAs and vsiRNA in SMV-infected soybean. **A)** Abundance and size distribution of total sRNAs from 18 to 30 nt in the 5 constructed libraries. Values are means + Sd of 2 biological replicates. One-sided Student’s *t* tests were used to determine significant differences at *P* < 0.05 (*). **B)** Abundance and size distribution of vsiRNA between 18 and 30 nt in the 5 constructed libraries. Statistical analysis was carried out as described in the legend of Fig. 1. Black and red asterisks indicate significant increase and decrease, respectively, at *P* < 0.05. **C)** Percentage of total sRNA in plants infected SMV-G7H::eGFP, SMV-G7H::eGFP::CPL, SMV-G7H::eGFP::CPS, or SMV-G7H::eGFP19 infected plants compared with healthy plants, and percentage of vsiRNA in their correspondent sRNA lines. **D)** Stem–loop qPCR for 2 vsiRNAs from the eGFP (siGFP) and the CI (siCI) regions to confirm vsiRNA-Seq analysis. SnoRI was used as an internal control. Statistical analysis was carried out as described in the legend of Fig. 1; * and ** indicate a significant difference at *P* < 0.05 and 0.01, respectively.

Changes in the levels of each sRNA species within every pool are expected due to viral infection. Thus, comparing total levels of sRNA among pools would not be meaningful. Instead, comparisons among treatments for each single sRNA and vsiRNA in the range of 18 to 30 nt were conducted to reveal any significant changes. Indeed, the distribution of sRNA in healthy plants showed that the 22-nt and 23-nt classes are predominant compared to the other sRNA classes. Interestingly, infection with SMV-G7H or its VSR-chimeras changed this profile by increasing the accumulation of 20-nt and 21-nt classes relative to other classes in almost all libraries (Fig. 4A and Supplemental Data Set S2).

In addition, a few other longer sRNA species (22-, 23-, 24-, 25-, 26-, and 27-nt classes) showed significant reduction primarily by G7H::eGFP::CPS (Fig. 4A). Regarding vsiRNAs, the 18-, 19-, 20-, 21-, and 22-nt classes were predominant following infection with G7H::eGFP, with the 21-nt class being the most abundant vsiRNA. VSRs, however, significantly repressed these predominant classes compared with G7H::eGFP (Fig. 4B), which indicates that these VSRs can deplete vsiRNAs available for RNA-induced silencing complexes. When calculating vsiRNAs percentage to their corresponding total sRNA pools, vsiRNA presented a small fraction ranging between 0.56% and 2.18% (Fig. 4C). The hotspots in the eGFP region (Supplemental Fig. S7) helped us design siRNA probes for the northern blot in *N. benthamiana* (Fig. 3A). But owing to their low accumulation levels in soybean, it was difficult to detect vsiRNAs by northern blot even when 3 different siRNA-eGFP probes were combined for detection. Instead, stem–loop quantitative PCR (qPCR) was used to confirm the siRNA-Seq data. Accumulation levels of 2 candidate vsiRNAs from the eGFP region (poolID324155) and the CI region (poolID15413), encoded in G7H, were significantly reduced in plants infected with the G7H::VSRs compared with G7H-eGFP (Fig. 4D). The data presented here confirmed the sRNA-Seq in which VSRs repressed the production of the vsiRNA.

### Genome mapping and vsiRNA hotspots

**Figure 5.**
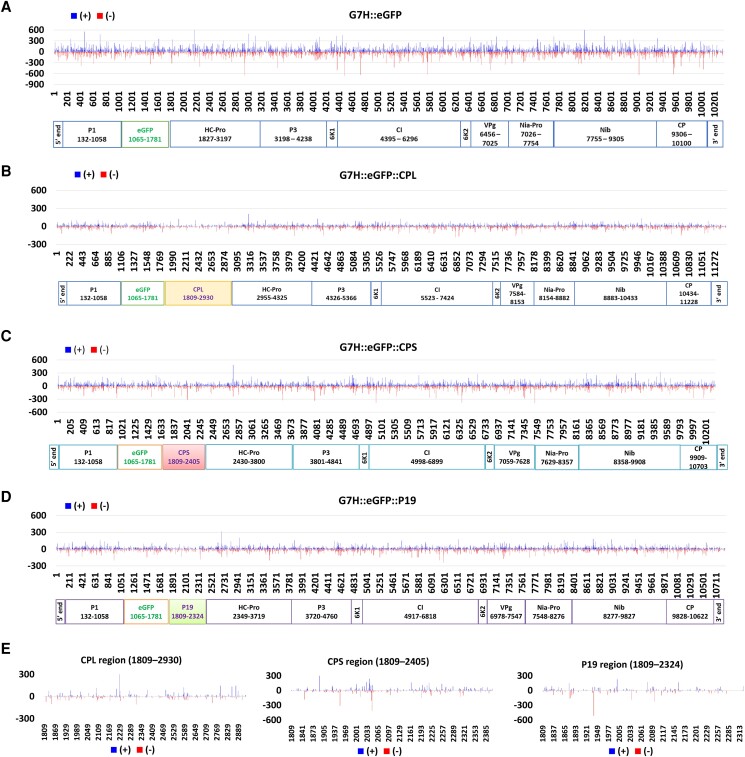
Distribution and RPM of vsiRNA along the SMV-G7H::eGFP genome and its chimeras. **A)** SMV-G7H::eGFP, **B)** SMV-G7H::eGFP::CPL, **C)** SMV-G7H::eGFP::CPS, **D)** SMV-G7H::eGFP::P19, and **E)** the specific regions of CPL, CPS, and P19 from **B** to **D)**, respectively. Upward blue and downward red denote the sense (+) and antisense (−) polarity reads, respectively. The genomic sites of SMV-encoded proteins are indicated below each panel.

To analyze vsiRNA origin across the viral genome, vsiRNA were located according to their 5′-end sites along both positive and negative strands of SMV-G7H::eGFP and its chimeras (Fig. 5, A to D, and Supplemental Data Set S3). All vsiRNAs are distributed almost continuously across the chimeras of SMV-G7H::eGFP. However, we detected several small regions where no vsiRNAs could be aligned (Fig. 5, A to D, and Supplemental Data Set S4). Reads for vsiRNA were lesser in the SMV-G7H::eGFP::VSRs than in the SMV-G7H::eGFP, and the hotspot maps were similar among the 4 variants except for the CPL, CPS, and P19 regions (Fig. 5E). In general, reads per million (RPM) were below 600 in SMV-G7H::eGFP (Fig. 5A) and became much less in SMV-G7H::eGFP::VSRs (Fig. 5, B to D). This indicates that these VSRs did not change the topology of the hotspot maps but did reduce the accumulation of the vsiRNA.

The 5′-end of the majority of the vsiRNAs have cytosine, which comprises around 42%∼45% of the vsiRNA compared with adenine (∼22%), guanine (16%∼20%), and uracil (15%∼16%) (Supplemental Fig. S6C). The pattern of 5′ composition did not change according to the vsiRNA size, with cytosine being prevalent in the 5′ end of the 20 nt and 21 nt dominant classes (Supplemental Fig. S6D). We observed asymmetrical distribution of vsiRNAs in sense-strand polarity in most of the libraries except for CPL, where the percentage of the antisense strand was slightly higher than that of the sense strand (Supplemental Fig. S6E). Altogether, these data indicate that these VSRs suppressed the production of vsiRNAs, and altered the sRNA profile in soybean plants.

### vasiRNAs targeting defense-related plant genes

**Figure 6.**
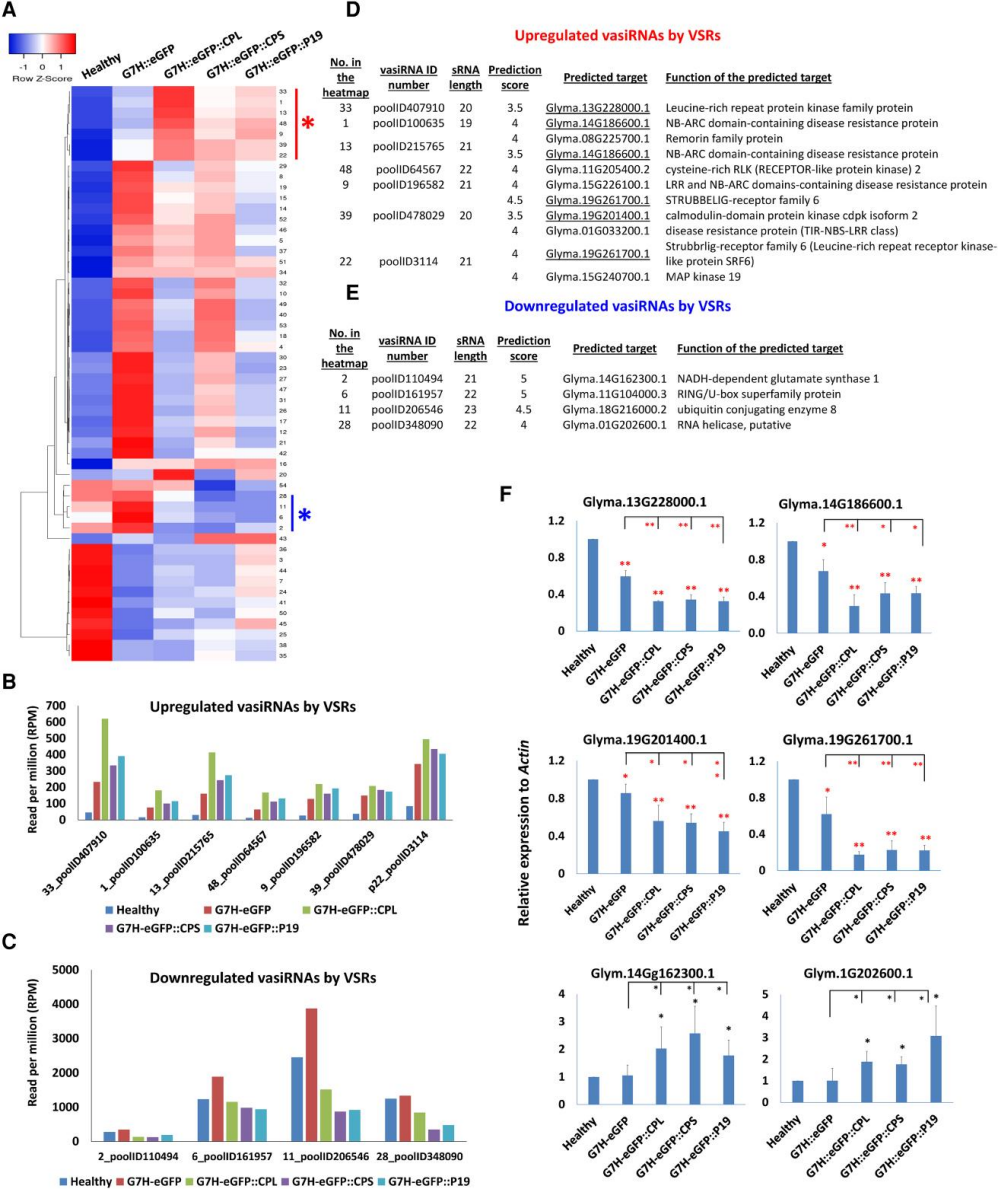
Commonly regulated vasiRNAs by CPL, CPS, and P19 and their potential targets in ‘L29’ plants. **A)** A heatmap of vasiRNAs (in RPM) extracted from sRNA libraries from healthy plants or those infected with G7H-eGFP or its variants. vasiRNAs with RPM less than 5 were removed. The red and blue asterisks indicate up- and downregulated vasiRNAs in CPL, CPS, and P19 chimeras. Single linkage was used as the clustering method, and the Pearson method was chosen for distance measurement. **B)** RPMs of the commonly increased vasiRNAs by VSRs. **C)** RPMs of the decreased vasiRNAs by VSRs. **D)** Target prediction of the increased vasiRNAs. **E)** Target prediction of the decreased vasiRNAs. **F)** qPCR of 6 selected target genes: Glyma.13G228000.1, Glyma.14G186600.1, Glyma.19G201400.1, Glyma.19G261700.1, Glyma.14G62300.1, and Glyma.1G202600.1. *Actin* was used as an internal control. Data are the average of 3 biological replicates. Values are means + Sd of 3 biological replicates. Statistical analysis was carried out as described in the legend of Fig. 1. * and ** indicate a significant difference at *P* < 0.05 and 0.01, respectively. Red and black asterisks indicate significant increase and decrease, respectively. The complete list of target genes is in Supplemental Table S5.

We next analyzed the potential of virus-activated siRNAs (vasiRNAs) to target defense-related genes. To identify putative targets, soybean vasiRNA targets were predicted by the fast algorithm of the TAPIR web server. After removing vasiRNAs of low RPM (below 5) and low target-prediction score (below 3.5), 54 vasiRNAs were obtained (Supplemental Data Set S5). Heatmap clustering showed a group of 7 vasiRNAs commonly induced by these VSRs, and another group of 4 that were similarly decreased, compared with SMV-G7H::eGFP or healthy plants (Fig. 6A). The RPMs ranged between 200 and 600 RPM for the upregulated vasiRNAs (Fig. 6B) but had a more comprehensive range for the downregulated ones (100 to 1,500 RPM) (Fig. 6C). To confirm these readings, stem–loop qPCR was carried out for 4 vasiRNAs, which showed a similar accumulation pattern to that obtained by sRNA-Seq reading (Supplemental Fig. S8).

Target prediction of vasiRNAs revealed several NB-LRR-type and kinase-related genes targeted by the upregulated vasiRNAs (Supplemental Data Set S5). In particular, LRR protein kinase (Glyma.13G228000.1), NB-ARC-related gene (Glyma.14G186600.1), calmodulin-dependent protein kinase 2 (Glyma.19G201400.1), the LRR receptor protein kinase Strubbelig-receptor 6 (Glyma.19G261700.1), and MAPK19 (Glyma.15G240700.1) were identified as potential targets for the upregulated vasiRNAs (Fig. 6D). Many of the target genes of the downregulated vasiRNAs were not related to defense pathways but were involved in different processes such as sugar transport, RNA synthesis, and protein degradation (Fig. 6E and Supplemental Data Set S5). qPCR analysis revealed that targets of the upregulated vasiRNAs (namely *Glyma.13G228000*.1, *Glyma.14G186600.1*, *Glyma.19G201400.1*, and *Glyma.19G261400.1*) were downregulated in G7H-eGFP::VSRs (Fig. 6F). In addition, targets of the downregulated vasiRNAs were upregulated in the G7H-eGFP::VSRs (*Glyma.14G162300.1* and *Glyma.01G202600.1*; Fig. 6F). Notably, 3 vasiRNAs (Nos. 6, 11, and 28) share the core sequence “CCCAGTCCCGAACCCGTCGGC,” which explains the similar targets for these vasiRNAs. In summary, several NLR and kinase-related genes were targeted by the upregulated vasiRNAs, which implies that the VSRs interfere with immune pathways on different levels.

### Early immune response in compatible and incompatible interactions for ‘L29’ plants against BPMV and SMV-G5H

**Figure 7.**
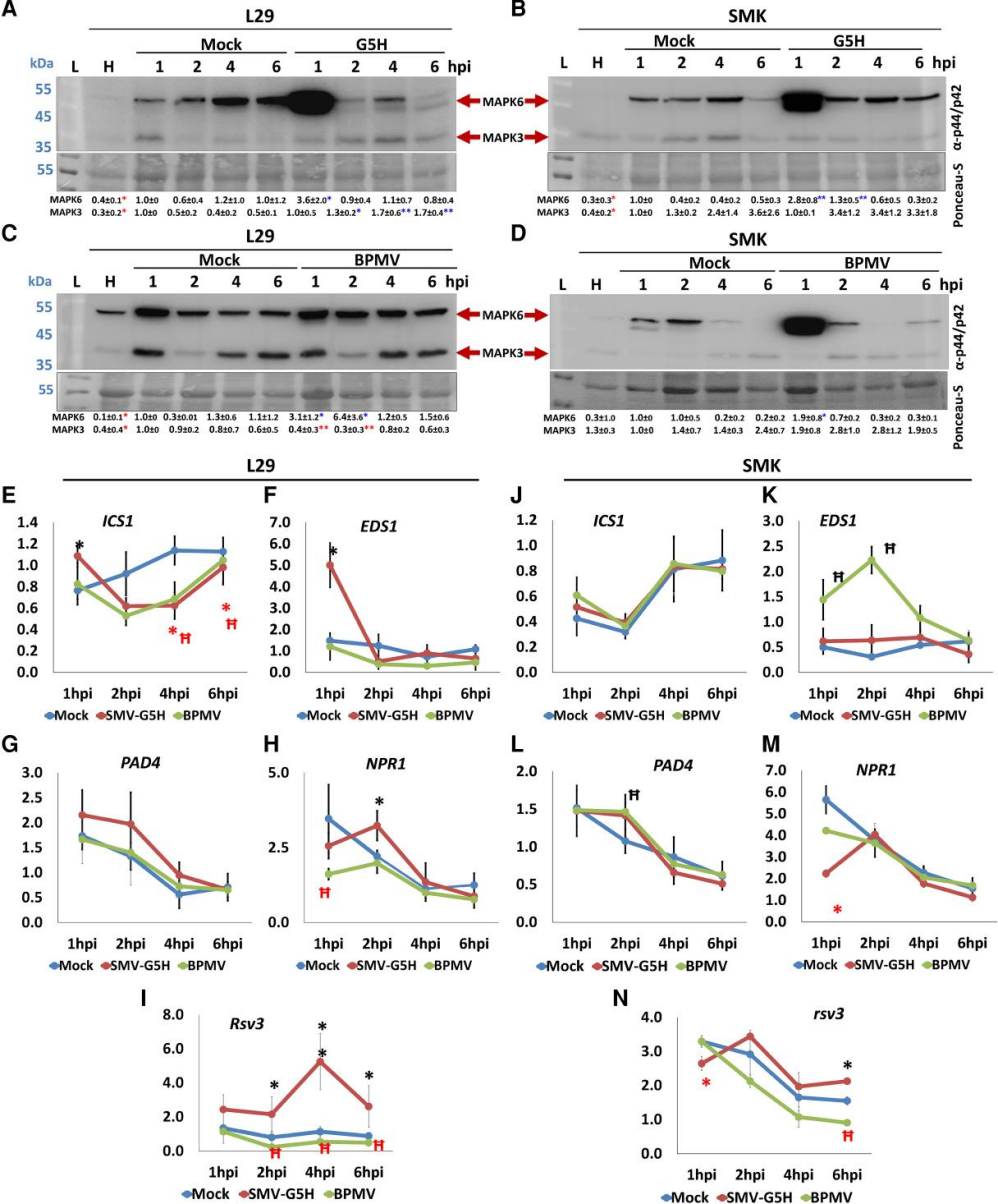
Early immune responses in ‘L29’ and ‘SMK’ plants infected with SMV-G5H or BPMV. **A** to **D)** Immunoblotting to determine the kinase activity of MAPK6 and MAPK3 in ‘L29’ plants infected with G5H strain of SMV SMV-G5H **A)** or with BPMV **C)**, and in ‘SMK’ plants infected with SMV-G5H **B)** or BPMV **D)**, at 1, 2, 4, and 6 dpi. The phospho-p44/42 MAP Erk1/2 antibody, which recognizes phosphorylated MPK3, MPK4, and MPK6 across kingdoms, was used to detect the kinase activities in soybean. Ponceau-S was used as an internal control. Blots represent 1 of 3 biological replicates. The numbers below the panels are the quantification of the bands relative to the RubScL level revealed by Ponceau-S and quantification was carried out by ImageJ. **E** to **M)** Relative expression levels of key genes in the SA pathway in ‘L29’ or ‘SMK’ leaves inoculated with SMV-G5H::eGFP or BPMV::GFP, respectively, for *ICS1***E**, **J)**, *EDS1***F**, **K)**, *PAD4***G**, **L)**, *NPR1***H**, **M)**, and *Rsv3* and *rsv* genes **I**, **N)**, and qPCR primers were designed based on the conserved regions between *Rsv3* and *rsv* CDS. Samples from healthy uninfected plants were collected at 0 hpi, and expression levels for all genes were measured at 1, 2, 4, and 6 hpi and normalized to that in healthy plants. Values are means ± Sd of 3 biological replicates. Statistical analysis was carried out as described in the legend of Fig. 1; * or Ħ indicate a significant difference at *P* < 0.01 or 0.05, respectively. * or Ħ indicate significant difference for G5H or BPMV, respectively. Black or blue symbols indicate significant increases, and red symbols indicate significant decreases.

Targeting several defense-related MAPK genes by vasiRNAs and the ability of BPMV to render plants susceptible to SMV infection promoted us to investigate whether BPMV may also affect the MAPK activity in soybean. MAPK3 and MAPK6 were previously reported to have proimmune and pro-SMV effects, respectively, in susceptible soybean plants (Liu et al. 2014; Xu et al. 2018). To address this, early immune responses were analyzed in BPMV- or G5H-infected plants. The resistant ‘L29’ cultivar responded to G5H infection by activating MAPK3 at all tested time points, while MAPK6 was only activated at 1 hpi and then declined afterward (Fig. 7A). In the susceptible G5H-infected ‘SMK’ plants, however, the activities of MAPKs were opposite, with MAPK6 activated at all time points, and the activity of MAPK3 decreased over time (Fig. 7A). Similarly, where the interaction between BPMV and ‘L29’ or ‘SMK’ plants is compatible, MAKP6 was activated in response to BPMV in both cultivars but lasted longer in ‘L29’ plants (Fig. 7, C and D). MAPK3 activity, however, was similar to that observed in mock plants in both ‘L29’ and ‘SMK’ plants (Fig. 7, C and D). Thus, BPMV might promote soybean susceptibility to SMV infection by inducing MAPK6.

Because MAPKs are affected by the SA pathway, we next analyzed the expression levels of the SA-related genes and the *Rsv3* gene in the presence of BPMV and G5H. In general, temporary increases in expression were observed for *ICS1*, *EDS1*, and *NPR1* following G5H infection on ‘L29’ plants (Fig. 7, E, F, and H). In contrast, BPMV infection decreased the expression of *ICS1* and *NPR1* (Fig. 7, F and H), and *PAD4* was not affected by either infection (Fig. 7G). The *Rsv3* gene was induced after G5H infection peaking at 4 hpi but significantly decreased with BPMV infection (Fig. 7I). On the other hand, ‘SMK’ plants showed almost no regulation in SA genes in response to G5H infection except for a temporary decrease in the expression of *NPR1* at 1 dpi (Fig. 7, J to M). BPMV infection increased the expression of *EDS1* and *PAD4* up to 2 hpi (Fig. 7, K and L). We then sequenced *rsv3* from ‘SMK’ plants. The amino acid sequence coding for the *rsv3* gene in ‘SMK’ plants had several deletions and substitutions within the CDS region relative to the *Rsv3* gene from ‘L29’ plants. Still, it was highly similar to the *rsv3* sequence in W82 plants (Supplemental Fig. S9A). This explains why rsv3 from ‘SMK’ clustered closer to that from W82 than Rsv3 from ‘L29’ in the phylogenetic tree (Supplemental Fig. S9B). Expression of the *rsv3* increased at 6 hpi following SMV-G5H infection and decreased at the same time following BPMV infection (Fig. 7N). Expression of ABA-related genes did not show significant changes in their expression at any time point for either virus in ‘L29’ plants (Supplemental Fig. S10). But in ‘SMK’ plants, a few *ABA* genes were downregulated at 4 and 6 h after infection with either virus (Supplemental Fig. S11). Collectively, these data indicate that in the incompatible interaction (G5H vs. ‘L29’), Rsv3 is induced at an early stage of infection, followed by MAPK3 activation and induction of SA genes. In compatible interactions, however, whether with ‘L29’ or ‘SMK’ plants, the activation of MAPK6 is apparent, and no SA gene was strongly induced in these conditions. BPMV infection induced MAPK6 in ‘L29’ plants, which, along with its encoded VSRs that suppress RNA silencing, increases the susceptibility of ‘L29’ plants to infection with G5H.

## Discussion

The *Rsv3* resistance gene provides soybean with a robust asymptomatic complete resistance against SMV-G5H because this strain cannot infect and reproduce in *Rsv3*-containing plants (Seo et al. 2014). It is an example of durable *R* gene resistance, a cornerstone of disease management in agriculture. Although viral synergism increases the impact of either or both viruses on the plant host, all reported cases described synergism in compatible interactions where the 2 unrelated viruses can naturally infect the same host (Syller and Grupa 2016; Pai et al. 2020). No study has described how 1 virus can suppress highly specific resistance against another virus. Our study revealed the molecular mechanism of impairing a durable *R* gene resistance via viral synergism.

BPMV synergizes with SMV-G5H by interfering with the downstream part of Rsv3 resistance, therefore locally suppressing the highly specific Rsv3 resistance against G5H in infected leaves. This suppression was mediated by BPMV-encoded VSRs (CPL and CPS) and occurred despite the induction of SA and RNA silencing-related genes. Both proteins exhibited VSR activities similar to those reported for P19 (Kontra et al. 2016; Garnelo Gomicronmez et al. 2021). CPL and CPS sequestered siRNAs and decreased the accumulation of vsiRNA (Figs. 2 and 3), thus explaining the increased virulence of SMV-G5H in soybean plants (Supplemental Figs. S3 and S4). This suggests that the effect of these VSRs is posttranscriptional and that the increased expression levels of SA and the RNA-silencing genes may not be an indication of primed resistance when mixed infections occur.

The ability of BPMV to suppress only local Rsv3-mediated resistance suggested that BPMV impaired 1 part of Rsv3-mediated ER but did not affect defenses pertained to systemic resistance. Several *R* genes were reported to have pleiotropic effects on resistance; impairing 1 component in the *R*-gene cascade compromises 1 aspect of resistance. The NLR Sw-5, for example, regulates 2 different resistance mechanisms against tomato spotted wilt virus (TSWV), i.e. (i) HR response regulated by the cytoplasmic function of Sw-5 and (ii) resistance pertaining to the intercellular movement, which is regulated by nuclear localization of Sw-5. Impairing these mechanisms allows TSWV to accumulate locally or spread systemically (Chen et al. 2021). Similarly, the Rsv1-mediated ER is controlled by different NLR resistance genes at the complex of the *Rsv1* locus, which offers different types of resistance. ER was maintained in soybean lines carrying the *3gG2* member of the Rsv1-associated subfamily. However, replacing *3gG2* with a set of 5 other NLR genes belonging to the same family compromised Rsv1-mediated resistance and allowed limited replication at the inoculation site (Wen et al. 2013). The *Ry_sto_* gene in potatoes confers HR or ER that depends on the load of the viral effector and the genetic background of the host but not on the expression levels of *RY_sto_* (Grech-Baran et al. 2020).

When ‘L29’ plants were fully infected with BPMV or SMV-G7H::VSRs, SMV-G5H::GUS could only accumulate in the inoculated leaves (Figs. 1 and 4) despite the ability of P19 to impair the systemic silencing signals (Yang et al. 2018). We previously showed that Rsv3-plants restricted SMV-G5H::GUS at the initial point of infection due to callose accumulation and that infected cell was cleared from viral RNA quickly without developing HR symptoms (Seo et al. 2009a, b, 2014). In this study, the SMV-G5H infection was initiated by sap inoculation on BPMV or SMV-G7H::VSRs-infected plants. It is evident in this situation that SMV-G5H replication and cell-to-cell movement can occur, but some aspects of phloem transport are blocked. This was evident because BPMV facilitated SMV-G5H movement outside infection foci on the same leaf but could not help facilitate systemic movement (Fig. 1, E and H). It can be suggested that BPMV did not need to evolve against the specific anti-G5H systemic resistance but has adapted well against the broad-spectrum antiviral RNA-silencing pathway; hence was unable to break part of the anti-G5H resistance.

The continuous accumulation of G5H in the inoculated leaves could be the reason behind the increased expression of *Rsv3* (Fig. 1G) and the activation of the RNA-silencing pathway, which is later impaired by the VSR activity of CPL, CPS, or P19 and thereby impairing the local resistance (Figs. 2 and 5). Interestingly, an *Rsv3-homolog*, *Rsc4-3* in cultivar ‘Dabaima’, is a cell-wall NLR that recognizes SMV-CI in the apoplast and then triggers anti-SMV defenses (Yin et al. 2021). Because this interaction occurs in the apoplast, the alert signal is transduced to the cell to produce proper defenses, where the VSRs encoded in BPMV or G7H::VSRs subsequently suppress these defenses to facilitate G5H infection of *Rsv3*-containing plants.

Activated ER may also target SMV-G7H because expression of CP from G7H in the G7H::VSRs single infection, where *Rsv3* was downregulated, was at least 6-fold higher than that in G7H alone. But after G5H::GUS infection, *Rsv3* increased and *CP* fold change for G7H::VSRs was only ∼2-folds higher than that in G7H alone (Fig. 3, F and G). Considering that G7H::GFP infection was systemically established when G5H::GUS was inoculated, the effect on SMV-G7H systemic resistance is undistinguishable.

Similar to our findings (Fig. 4), all the sRNA size classes between 20 and 25 nt are enriched in soybean plants (Zheng et al. 2016; Jia et al. 2020; Wang et al. 2020). However, SMV infection enhanced the 20 nt and 21 nt compared to other sRNA sizes (Fig. 4A), which is probably the reason for the increased levels of vasiRNAs (Fig. 4B). These vasiRNAs were found to target several kinases and NLR-type defense-related genes (Fig. 6D and Supplemental Data Set S5), including the receptor-like kinase Strubbelig-receptor 6 (*SUB6*), whose mutant caused growth abnormalities in *Arabidopsis thaliana* (Chevalier et al. 2005). The suppressed expression of *SUB6* by SMV-G7H::VSRs may explain the abnormalities in soybean plants infected with BPMV or SMV-G7H::P19 (Figs. 3D and S1B). The calmodulin-dependent protein kinase, which regulates immune responses to a few viruses (Perraki et al. 2018), was downregulated in G7H::VSRs, probably via vasiRNA (Fig. 6C). Although callose accumulation is part of the Rsv3-mediated resistance against SMV-G5H (Seo et al. 2014), we could not detect any vasiRNA targeting genes from the callose synthase family or β-1,3 glucanase family. This implies that if BPMV affects callose, such effect could be at the transcriptional or posttranscriptional levels or mediated by other BPMV proteins.

The roles of MAPK in early immune responses are well established and include positive functions for MAPK3 and MAPK6 in *Arabidopsis* (Lang and Colcombet 2020). In soybean, however, silencing MAPK6 rendered plants more resistant to infection with SMV (Liu et al. 2014). Our data showed that in all compatible interactions (‘L29’ vs. BPMV and ‘SMK’ vs. SMV-G5H or BPMV), MAPK6 was strongly activated throughout the assay. In contrast, activity did not change (in ‘L29’) or even reduced (in ‘SMK’) in these interactions (Fig. 7, B to D). However, in the lone incompatible interaction example (‘L29’ vs. SMV-G5H), MAPK3 exhibited persistent activation associated with reduced MAPK6 activity (Fig. 7A). The early increased expression of *Rsv3* in this interaction (Fig. 7I) suggests that MAPK3 may have a role in *Rsv3*-mediated ER. The activation of MAPK6 in compatible interactions also supports the inference that it has negative roles in soybean defense. MAPK4 could not be detected in soybean following infection with either virus, which is in line with a previous finding that the separate application of SA or flagellin failed to induce MAPK4 in soybean but did induce MAPK6 (Xu et al. 2018).

We previously found that *Rsv3* was not induced at the later stage of G5H infection in ‘L29’ plants, probably because the fold change set in the RNA-Seq analysis was at least 1-fold (Alazem et al. 2018). It appears that *Rsv3* is strongly induced at the very early stage of incompatible infections for G5H (Fig. 7I) or G7 (DeMers et al. 2020), peaking at 4 hpi, which was also accompanied by induction of SA-related genes (Fig. 7). However, in the compatible interactions between BPMV with either ‘L29’ or ‘SMK’ (Figs. 7 and S2) or G7H with ‘L29’ (Alazem et al. 2018), SA-related genes were induced at the later stage of infection. This strongly indicates that increased stress in compatible interactions leads to the later activation of SA. Such activation can be more robust in plants carrying anti-SMV *Rsv* genes than in *rsv*-plants.

Impairing durable *R* gene resistance by viral synergism, such as the BPMV-SMV example, is a sign of possible disease outbreaks. While BPMV may not affect the Rsv3 recognition of the G5H-CI effector, this virus offers synergism to G5H by interfering with the downstream part of this Rsv3-resistance by decreasing the expression of Rsv3, impairs the RNA-silencing pathway via its VSR proteins, and thus impairs the highly specific ER against G5H in the infected leaves. This demonstrates how viruses target the actual defense mechanisms in the cascade downstream of the *R* gene and that recognizing viral effector, and thus triggering the defense signaling cascade, may not be effective when the synergizing virus directly impairs downstream defenses.

## Materials and methods

### Plasmid construction

The plasmids pSMV-G5H::eGFP, pSMV-G5H::GUS, and pSMV-G7H::eGFP were previously constructed (Seo et al. 2009a, b). CPL and CPS subunits from BPMV RNA 2 (V2) and P19 were cloned downstream of the eGFP gene in the infectious clones pSMV::G5H and pSMV::G7H (Supplemental Fig. S1C). The infectious clone of the bipartite BPMV comprises 2 plasmids, R1 and V2 (or V2 expressing GFP), that were constructed by Zhang et al. (2010). At 12 d postinfection (dpi), the 2nd trifoliate leaves were collected for RNA extraction and gene expression analysis.

### Plant materials, growing conditions, and virus infections

The SMV-susceptible soybean (*G. max*) cultivars ‘Lee74’ (*rsv*) and Somyongkong (‘SMK’; *rsv*) (Kim et al. 2003; Khatabi et al. 2012) and the SMV-G5H-strain-specific resistant cultivar ‘L29’ (*Rsv3*), which is a near W82 isoline carrying *Rsv3* from Hardee (Gunduz et al. 2001), were grown in growth chambers at 25 °C with 70% relative humidity and a 16/8 h photoperiod. Plants were inoculated with infectious sap, as described previously (Alazem et al. 2018). Plants infected at the unifoliate stage were grown for another 12 d before they were further infected with G5H or G7H. For BPMV infection, 10 *μ*g of R1 and V2 plasmids (or V2 with GFP) was rub-inoculated on the 1st unifoliate leaves as described previously (Zhang et al. 2010). At 12 dpi, BPMV-only infected samples were collected from the second trifoliate leaves; the same leaves of the same plants were then sap-inoculated with SMV-G7H::eGFP, SMV-G5H::eGFP, or their chimeras expressing CPL, CPS, or P19. Samples from mixed infections were collected at 5 and 10 d post the second infection for the inoculated leaves and at 10 d for the systemically infected leaves. Sampling for all experiments, except the RNA-Seq experiment, was carried out on 3 biological replicates. Each replicate consisted of 3 plants, and 1 trifoliate leaf was collected from each plant, resulting in a total of 9 singular leaves per biological replicate.

### RNA extraction and qPCR

Total RNA was extracted by the TRIzol (Sigma) method following the manufacturer's instructions. RNA quality was assessed by Nanodrop, and samples with a 260/280 ratio of ∼2.1 and a 260/230 ratio of ∼2.0 were used for cDNA synthesis. One microgram of total RNA was used for cDNA synthesis with the GoScript kit (Promega, USA). qPCR was carried out with SYBR-Green (Promega) to measure the relative expression of target genes using the ΔΔCT method and the BioRad CFX Connect qPCR Detection System. *Actin11* was used as an internal control for gene expression, and GmSnoRI was used as an internal control for the stem–loop qPCR. Experiments were run in 3 biological replicates, each of which consisted of 3 technical replicates. Quantification cycle (*C*_q_) values for *Actin11* were ∼20 to 21, and for the plant, genes were ∼25 to 31. Primers used in this study are listed in Supplemental Table S1.

### Virus-induced gene silencing of RDR1a and RDR6a

To silence *RDR1a* and *RDR6a* in ‘L29’ plants, fragments of ∼540 and 650 bp were amplified from ‘L29’ plants and then cloned in the multiple cloning site of RNA2 of BPMV, as described previously (Zhang et al. 2010; Bwalya et al. 2022). Ten micrograms of BPMV plasmids (RNA1 and RNA2) were rub-inoculated onto the first unifoliate leaves of ‘L29’ plants, and the silencing efficiency was tested at 14 dpi in the second trifoliate leaf. The same second trifoliate leaf was sap-inoculated with G5H::GUS, and samples were collected from the inoculated leaves 5 dpi for further analyses.

### Small RNA library and bioinformatics analyses

A pool of 9 leaves from 3 plants was mixed and portioned into 0.1 g portions for RNA extraction. For library construction, Next Generation Sequencing (NGS) of sRNA was carried out by the HiSeq2500 system using SMARTer smRNA-Seq Kit for Illumina with the single-end type of reading for sRNAs between 18 and 50 bp (Macrogen, South Korea). Ten libraries were generated from 2 biological replicates. For quality filtering and to remove poly-A-tailing adapters, the raw data were processed by Cutadapt 3.4 (Martin 2011) with the command line “cutadapt -a AAAAAAAAAA -O 5 --max-n=0 -m 18 -n 2 -q 10 -u 3”. Customized Perl scripts were used to remove poly-A/T/C/G stretches and to group the clean reads into unique reads (tags) by sequence identity. Low-frequency tags and tags with sizes <18 nt or >30 nt were filtered out. Total sRNAs were mapped to the soybean genome (W82 a4. V1). Identification of virus-derived siRNAs (vsiRNAs) was done by aligning against the SMV-G7H::eGFP genome expressing CPL, CPS, or P19 via perfect match. sRNA abundance was normalized into reads per million (RPM) to compare sRNA expression among libraries. To identify putative soybean targets specifically regulated by SMV infection, soybean vasiRNA targets were predicted by the fast algorithm of the TAPIR web server (Bonnet et al. 2010), with a score cut-off = 4. FASTA files of the short-listed vasiRNAs and soybean transcriptome were uploaded to the servers for calculation. Heatmap was generated by Heatmapper (Babicki et al. 2016). sRNA-Seq raw data were deposited in Sequence Read Archive (SRA) on NCBI under accession number PRJNA761550.

### Stem–loop RT–PCR

The stem–loop RT–PCR was carried out as described previously (Varkonyi-Gasic et al. 2007; Yu et al. 2018). In brief, low molecular weight (LMW) RNA was extracted with a mirVana isolation kit (Ambion, USA) following the manufacturer's instruction. 0.5 *μ*g of LMW was denatured at 65 °C for 5 min and then chilled on ice. Pulsed RT and end-point PCR (and qPCR) reactions were carried out as described previously (Yu et al. 2018). In brief, pulsed RT was conducted by specific primers targeting specific sRNA generated from eGFP and CI regions (siGFP and siCI) using SuperScript III reverse transcriptase (Invitrogen, USA). The end-point PCR was carried out with 200 ng of RT mixture to amplify a 21-bp sense siRNA-GFP and -CI (poolID324155 and poolID15413, respectively). The final PCR products were visualized on a 4% *w*/*v* agarose gel.

### Protein blot

Total protein was extracted from 0.1 g of tissue collected from a pool of inoculated or systemically infected leaves from 3 plants, as described previously (Alazem et al. 2018). Constructs expressing eGFP or GUS were detected by protein blot using polyclonal anti-GFP or anti-GUS antibodies (Sigma). All primary antibodies were bound with the goat anti-rabbit secondary antibody (Cell Signalling). Ponceau-S was used as a loading control.

### Protein and RNA coimmunoprecipitation

Transient expression and co-immunoprecipitation were carried out as described previously (Alazem et al. 2017). *N. benthamiana* leaves were collected 2 d after coagroinfiltration with pBin-3HA-mCherry expressing either CPL, CPS, or P19 (in the C-terminal mCherry protein tag) along with pBin-eGFP. Total protein was extracted from 2 g of leaves (6 leaves from 3 plants) in extraction buffer (20 mM Tris–HCl, pH 7.5, 150 mM NaCl, 5 M MgCl_2_, 5 mM DTT, and 5% *v*/*v* Nonidet P-40) with protease inhibitor cocktail (Roche). Immunoprecipitation was carried out as described previously (Alazem et al. 2017). For input samples, HA was detected using anti-HA antibodies (clone 3F10; Roche). For co-immunoprecipitated samples, mCherry was detected using an anti-mCherry antibody. Immunoprecipitated RNA was extracted from HA-beads by TRIzol, as described previously (Brosseau and Moffett 2015). An amount of 250 ng of precipitated RNA was used to detect siGFP by stem–loop PCR, as described above.

### Northern blot

sRNA was extracted from *N. benthamiana* leaves with a mirVana isolation kit. Northern blot for sRNA was carried out, as described previously (Alazem et al. 2017). In short, an amount of 5 *μ*g of sRNA was run in 19% acrylamide/7 M urea gel, transferred onto a Hybond-N+ membrane (Life Technology), cross-linked under UV light, and hybridized against 32P-labeled (−)siGFP or U6 probes (Supplemental Table S1).

### MAPK assay

‘L29’ and ‘SMK’ plants were inoculated with BPMV::GFP or SMV-G5H::eGFP sap extracted from infected ‘Lee74’ plants, as described earlier. Mock plants were treated with phosphate buffer. At 30 to 40 min after inoculation, leaves were washed with water, and samples were collected at 1, 2, 4, and 6 h post inoculation (hpi). Phosphorylation activity of MAPK6 and MAPK3 was detected by hybridizing protein blots with Phospho-p44/42 MAPK (Erk1/2) (Thr202/Tyr204), which recognizes phosphorylated MPK3, MPK4, and MPK6 across kingdoms, and anti-rabbit IgG second antibody (Cell Signalling).

### Phylogenetic analysis

The Mega X software was used to carry out the phylogenetic analysis using the neighbor joining method. Phylogenetic tree was constructed based on the amino acid (aa) sequences of Rsv3 from ‘L29’, rsv from W82 and ‘SMK’, and the *N. benthamiana* disease resistance NLR protein (Niben101Scf01494g09005.1) which is the closest NLR to Rsv3 in the *N. benthamiana* database.

### Accession numbers

Original sRNA-Seq data are available at the NCBI Sequence Read Archive (SRA) under accession code PRJNA761550.

## Supplementary Material

kiad255_Supplementary_Data

## Data Availability

The data that support the findings of this study are available from the corresponding authors upon reasonable request. Restrictions may apply to the availability of these data, which were used under the Creative Commons CC BY license for this study.

## Supplemental data

The following materials are available in the online version of this article.

**Supplemental Figure S1.** Experimental design, chimera constructions, and effect of prolonged infection with BPMV on ‘L29’ (Rsv3-plants) growth.

**Supplemental Figure S2.** Effect of BPMV on defense signaling pathways in ‘L29’ and Somyongkong (‘SMK’) plants.

**Supplemental Figure S3.** Chimera constructions and the effect of CPL, CPS, or P19 on G5H strain of SMV (SMV-G5H) virulence in ‘Lee74’ plants.

**Supplemental Figure S4.** Effect of the VSRs CPL, CPS, and P19 on G7H virulence in ‘Lee74’ plants.

**Supplemental Figure S5.** Effect of silencing *RDR1a* and *RDR6a* on Rsv3-mediated resistance to SMV-G5H-GUS.

**Supplemental Figure S6.** Characteristics of sRNAs and vsiRNA in SMV-infected soybean.

**Supplemental Figure S7.** siRNA eGFP distribution and levels in G7H::VSRs::eGFP backbones expressing CPL, CPS, or P19 from ‘L29’-infected plants.

**Supplemental Figure S8.** Stem–loop qPCR for selected vasiRNAs from Fig. 6A.

**Supplemental Figure S9.** Sequence alignment and phylogenetic analysis of Rsv3.

**Supplemental Figure S10.** Expression of ABA-related genes in ‘L29’ (Rsv3) plants infected with the G5H strain of SMV SMV-G5H or with BPMV.

**Supplemental Figure S11.** Expression of ABA-related genes in ‘SMK’ (rsv) plants infected with the G5H strain of SMV SMV-G5H or with BPMV.

**Supplemental Table S1.** Primers used in this study.

**Supplemental Data Set 1.** Overview and total reads of sRNA RNA-Seq from ‘L29’ plants infected with G7H::eGFP, G7H::eGFP::CPL, G7H::eGFP::CPS, and G7H::eGFP::P19.

**Supplemental Data Set 2.** Reads, the size distribution of total sRNAs, and unique tags (18 to 30 nt) of the constructed libraries from healthy ‘L29’ plants, or those infected with G7H::eGFP, G7H::eGFP::CPL, G7H::eGFP::CPS, and G7H::eGFP::P19.

**Supplemental Data Set 3.** vsiRNA mappings in G7H::eGFP, G7H::eGFP::CPL, G7H::eGFP::CPS, G7H::eGFP::P19, and in eGFP region for all constructs.

**Supplemental Data Set 4.** Sequences, lengths, reads, polarity, and mapping sites for vsiRNAs generated from G7H::eGFP, G7H::eGFP::CPL, G7H::eGFP::CPS, and G7H::eGFP::P19 genomes.

**Supplemental Data Set 5.** RPMs, reads, and target predictions for the 7 upregulated and 4 downregulated vasiRNAs from Fig. 6A.
