# Supplementary material for: Viral synergism suppresses R gene-mediated resistance by impairing downstream defense mechanisms in soybean
Source: Plant Physiol. 2023 Apr 26;192(4):3088–105. doi: 10.1093/plphys/kiad255 (PMC10400036; doi:10.1093/plphys/kiad255)
Supplement: kiad255_Supplementary_Data [file kiad255_supplementary_data.zip › Supplemental Data.pdf]

Viral Synergism breaks down *R*-gene-mediated extreme resistance by impairing downstream defense mechanisms in resistant soybean plants

Mazen Alazem[1,2], John Bwalya[3,] Pai Hsuan[4], Jisuk Yu[1], Huong Chu Cam[3], Tessa Burch-Smith[2], and Kook-Hyung Kim[1,3,5]

[1]Plant Genomics and Breeding Institute, Seoul National University, Seoul, Republic of Korea; [2]The Donald Danforth Plant Science Center, St. Louis, MO 63132, USA; [3]Department of Agricultural Biotechnology, College of Agriculture and Life Sciences, Seoul National University, Seoul, Republic of Korea; [4]The Sainsbury Laboratory, University of East Anglia, Norwich Research Park, Norwich, UK; [5]Research Institute of Agriculture and Life Sciences, Seoul National University, Seoul, Republic of Korea

Supplemental Table S1. Primers used in this study

Supplemental Table S1. Primers used in this study					
**No**	**Name**	**F-Seq**	**R-Seq**	**Accession No.**	**Purpose**
**1**	EDS1	CCTTGGTCAAGTGGTGGAAAAC	CACAGCCTAATGCATGTTTGTC	Glyma.06G187300	**RTqPCR**
**2**	ICS1	CAAGCTTGACTTGCTTCGGA	CGAGGGTAGGATACCCAAAGGA	Glyma. 03G070600	
**3**	NPR1	TGGAGGATGACTTGCCTGACT	GGCTAAACTCGGCCTTGTCC	Glyma.15G127200	
**4**	PAD4	GCTTTTCGTACTCGCTGGCT	TCCTCTTCCAGCCACTCCTG	Glyma.13g04540	
**5**	DCL2a	GGCGGTGCTCATAAGGACAC	ACCCTTGTGCACAGTACACA	Glyma.09G025400	
**6**	DCL4a	GAGGGACCAGACCACCTGAA	CCATAATGCACCCTCTGCCG	Glyma.13G156500	
**7**	RDR1a	TCCAAGTTACTGGGGTTGCT	ACGCAACCCACTGAAACTGT	Glyma.02G086100	
**8**	RDR1a for qPCR in VIGS)	GCACTCCCTATTTTCCAGTTGG	GCACACCCTCATTCCTTGATAG	Glyma.02G086100	
**9**	RDR2b	GGTAACGTGCAAAACCGTGC	ACGCGGTTGGCAACTAGTTT	GLYMA.17G091500	
**10**	RDR6a	CAGTTGATTACCTAGCTCGC	GAGCAAGCTCTCAATGGAAT	Glyma. 04G067300	
**11**	AGO1b	CTCACCTTGCTGCATTCCGT	TGCAACAGCACCACTTGTCA	Glyma.09G167100	
**12**	AGO3b	TGAAGCAAGGATCAGGATGCA	TCCAGGTCAGCATGCAATGT	Glyma. 15G126700	
**13**	AGO4b	TGGCTGCTACTCAGATGGGT	GCAGGCATACCAGAACCACC	Glyma. 02G274900	
**14**	AGO5b	AGGAACTAGTCGACCAACACA	TCGAGTACACCTTGCATACGT	Glyma. 11G190900	
**15**	AGO6a	TTGGTTTCTCGGCAGATGGC	GCAGCATGGTGAGCATAGCA	Glyma.13G193200	
**16**	AGO7b	ATTTCTTTGGTGCCTCCTGC	GGTGCTGTCTTTGGAGGAGC	Glyma.02G111600	
**17**	AGO9	TGGGTTCGCGGTATTGGTTG	TCTTCCTCCTCCTGCAACCC	Glyma.06G314500	
**18**	AGO10c	GCGCGGTTCTATATGGAGCC	TCTTGTTGCCTTGGAGCCAC	Glyma.02G002500	
**19**	ABA2_1400	CATGGTTGATGGAGGCTTCAC	ACCCTTGTACTAGACATCAGGA	Glyma.11G151400	
**20**	ABA2_1700	CATGGTTGATGGAGGCTTCAC	TTGTAATGTCTCAAACTGCAAACA	Glyma. 11G151700	
**21**	ABA1_4500	CGCATCCGTCCATCTGATGT	TCCCTGCAAAGCTAGTGTGC	Glyma.17G74500	
**22**	ABA1_5700	CGCATCCGTCCATCTGATGT	ACCTTAACACGGTATGAAGCCT	Glyma.11G055700	
**23**	AAO3_2200	AGGAACTCTGTGGACTGGAC	GGATAGTGATGAGCACCGTT	Glyma.02G272200	
**24**	AAO3_2400	AGGAACTCTGTGGACTGGAC	AGTTCCTTTACTTGTTGCCCATT	Glyma. 02G272400	
**25**	AAO3_5100	AGGAACTCTGTGGACTGGAC	AGTGAGCACCATTATGTCCTTC	Glyma. 14G45100	
**26**	Rsv3 and rsv3	GTGAATTGAACAAGGCATGTGG	TGGCAGTGACAAGGAGAGATA	Glyma14g204700	
**27**	SMV-CP (G 7H)	GCAATAGCGCAAATGAAGGC	TGCCCAAAAGAGTGTGCATG		
**28**	eGFP	AGCTGAAGGGCATCGACTTC	TTCTGCTTGTCGGCCATGAT		
**29**	Glyma.13G2 28000.1	GCTTGAGAGAGACTTGCCGT	GCATCCTCCTGTTCCAGTCC	Glyma.13G228000	
**30**	Glyma.14G1 86600	TCCGGATCTGTTCCAAAATGC	CCGGGTCCAGGAGTACATTG	Glyma.14G186600	
**31**	Gm19g2014 00.1 (CDP K)	ACGGATGACAGCACATGAAG	TTCAGGCGACTCAAAACTGC	Gm19g201400	
**32**	Glyma.19G2	GCGAGCTAACATGAGCAAGC	CCTCGTTGGGATCCTCCATG	Glyma.19G261700	

	61700.1				
**33**	Glym.14Gg 162300.1	TGCAACGTTCGAGATGCTTG	AGCAACAGCTTCTGCACTTC	Glym.14Gg162300	
**34**	Glyma.01g2 02600.1	TTCTCTTCTCTCTCCCCCTAAC	ATTAGGGTTAGGGTTGGGTGTG	Glyma.01g202600	
**35**	Actin11	ATCTTGACTGAGCGTGGTTATTCC	GCTGGTCCTGGCTGTCTCC	Glyma.18G290800	
**36**	RDR1a	gcGGATCCAAAtGAAATATCAACAAGTG	GCCTCGAGACTCAAACGCAACCC	Glyma.02G086100	VIGS
**37**	RDR6a	GCgaGGATCCaGTACATGCTGATTCCA	GCCTCGAGGCAGATCAAGGGATTTT	Glyma. 04G067300	VIGS
**38**	BPMV-MP	GTTCTTGCAAAGCGAGCTCA	TCGGAAATGTAACCACCCGA		RT-PCR
**39**	P19 for pS NU-G5H an d pSNU-G7 H	TTTACAGTCAACGCGTATGGAACGAGCT ATACAAGGAAACG	AGACAGACTCACGCGTCTCGCTTTCTTTTTCGA AGGTCTC		cloning into pSNU-S MV
**40**	CPL for pS NU-G5H an d pSNU-G7 H	TTTACAGTCAACGCGTATGGAAACAAATT TGTTTAAATTGT	AGACAGACTCACGCGTCTGAGGAATGGTGCCC AAAAGA		
**41**	CPS for pS NU-G5H or G7H	CAGACGCGTAGTCCATTTCACAGCAAACT	GCGACGCGTTGCAGAGGATTCCGCATT		
**42**	CPL_SalI	AGATTACGCTGTCGACATGGAAACAAAT TTGTTTAAATTGT	ATTCGAGCTCCCCGGGTCACTGAGGAATGGTG CCCAAA		Cloning into pBin-3H A by InFusion kit
**43**	CPS_SalI	AGATTACGCTGTCGACATGTCCATTTCAC AGCAAACT	ATTCGAGCTCCCCGGGTCATGCAGAGGATTCC GCA		
**44**	P19_SalI	AGATTACGCTGTCGACATGGAACGAGCT ATACAAGGAAACG	ATTCGAGCTCCCCGGGTCAGAAGGTCTCAGTA CCTTCAGGG		
**45**	pSNU G5/7 -GFP Check MCS	TCCTGCTGGAGTTCGTGAC	GGTGCATTCATGATTCTCCAC		Sequencing the insert s in pSNU-SMV
**46**	pBin61	CCCAACCACGTCTTCAAAGC	CCCTTATCGGGAAACTACTCA		Sequencing the insert s in pBin61-3HA
**47**	siRNA eGF P target (A CCCTGAA GTTCATCT GCACC)	gCggCggACCCTGAAGTTCATC	GTCGTATCCAGTGCAGGGTCCGAGGTATTCGC ACTGGATACGACGGTGCA		Stem-Loop RTqPCR
**48**	siRNA CI t arget CAA GGTGACA AAGGTTG ATGG	gCggCggCAAGGTGACAAAGGT	GTCGTATCCAGTGCAGGGTCCGAGGTATTCGC ACTGGATACGACCCATCA		
**49**	poolID1104 94 (CCTCA GTGGCGG ACCGGGC CC)	gCggCggTCCCAGTCCCGAACC	GTCGTATCCAGTGCAGGGTCCGAGGTATTCGC ACTGGATACGACAGCCGA		

**50**	poolID206546 (TCCCAGTCCCGAACCCGTCGGCT)	gCggCggTCCCAGTCCCGAACC	GTCGTATCCAGTGCAGGGTCCGAGGTATTCGCACTGGATACGACAGCCGA		
**51**	poolID100635 (TTCCACAGCTTTCTTGAAC)	gCggCggTTCCACAGCTTTC	GTCGTATCCAGTGCAGGGTCCGAGGTATTCGCACTGGATACGACGTTCAA		
**52**	poolID3114 (TGAAGCTGCCAGCATGATCTG)	gCggCggTGAAGCTGCCAGCAT	GTCGTATCCAGTGCAGGGTCCGAGGTATTCGCACTGGATACGACCAGATC		
**53**	URP (universal reverse primer)	---	gTgCAgggTCCgAggT		
**54**	SnoRI (Reference gene)	GAAGATGAAGAGCTTTGTATATTC	ACTCAGAGAGTTGCTTTCTGTG		
**55**	si-eGFP oligo-1	TTGCCGGTGGTGCAGATGAACTTCAGGGTCAGCTTGCCG	Designated based on Fig. S6		Northern blot
**56**	si-eGFP oligo-2	GTTGCCGTCGTCCTTGAAGAAGATGGTGCGCTCCTGGACG			
**57**	si-eGFP oligo-3	GTTGTGGCGGATCTTGAAGTTCACCTTGATGCCGTTCTTCTG			
**58**	Nb.U6	GCTAATCTTCTCTGTATCGTTCC			

Supplemental Figure S1

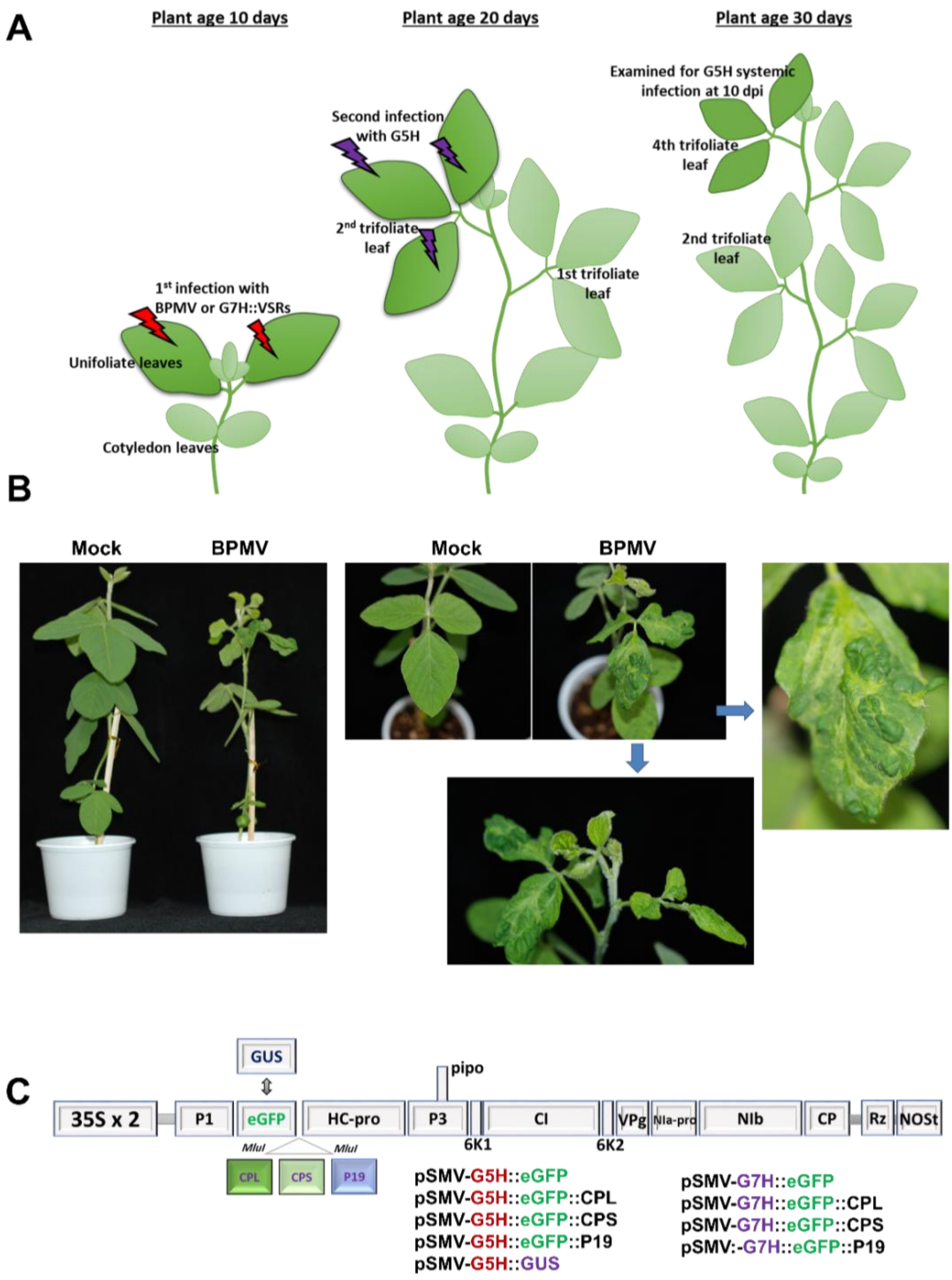

Supplemental Figure S1. Experimental design, Chimera constructions, and Effect of prolonged infection with bean pod mottle virus (BPMV) on L29 (Rsv3-plants) growth.

Supplemental Figure S2

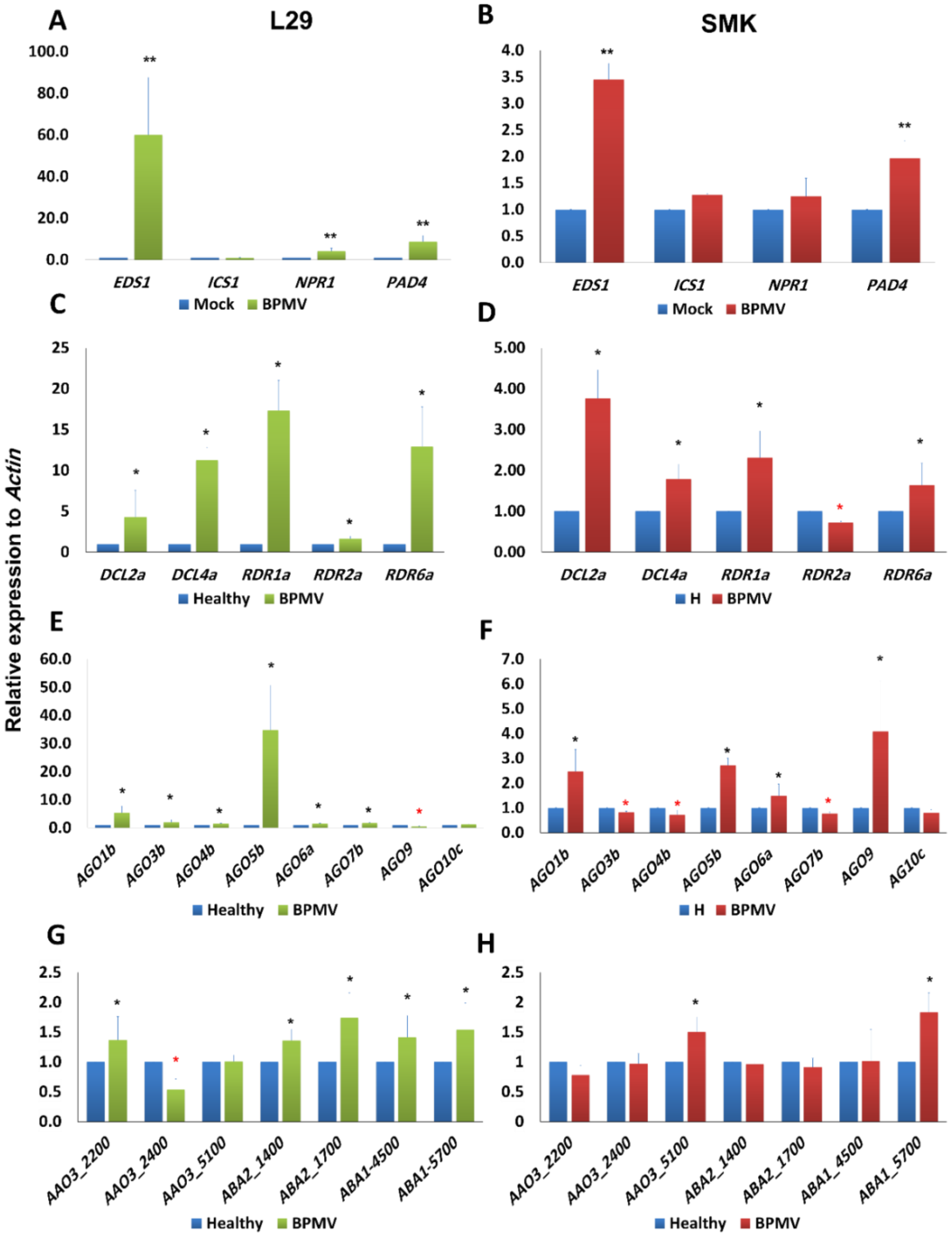

Supplemental Figure S2. Effect of Bean pod mottle virus (BPMV) on defense signaling pathways in L29 and Somyongkong (SMK) plants. Effect of BPMV on defense signaling

pathways on L29 and SMK plants. A-B, Relative expression levels, as determined by qPCR, for the SA-related genes Enhanced Disease Susceptibility1 (*EDS1*), Isochorismate Synthase1 (*ICS1*), Phytoalexin deficient 4 (*PAD4*), and Nonexpresser of Pathogenesis-Related genes 1 *(NPR1*) following infection with BPMV in L29 (A) and SMK (B) plants. C-D, Relative expression of the Dicer-Like (DCL) genes *DCL2a*, *DCL4a*, RNA-dependent RNA Polymerase (RDR) genes *RDR1a*, *RDR2a*, and *RDR6a* in L29 (C) and SMK (D) plants. E-F, Relative expression levels of Argonaute (AGO) genes (*AGO1b, 3b, 4b, 5b, 6a, 7b, 9*, and *10c*) in L29 (E) and SMK (F) plants. G-H, Relative expression levels of ABA biosynthesis genes (*AAO3_2200, AAO3_2400, AAO3_5100, ABA2_1400, ABA2_1700, ABA1_4500*, and *ABA1_5700*) in L29 (G) and SMK (H) plants. Samples were collected from the systemically infected leaves at 12 dpi for qPCR analyses, and *Actin11* was used as an internal control. Values are means + SD of three biological replicates. One-sided student *t*-tests were used to determine significant differences at $P < 0.05$ (*) and $P < 0.01$ (**) for gene expressions in healthy and BPMV-infected plants. Black and red asterisks indicate significant increase and decrease, respectively.

Supplemental Figure S3

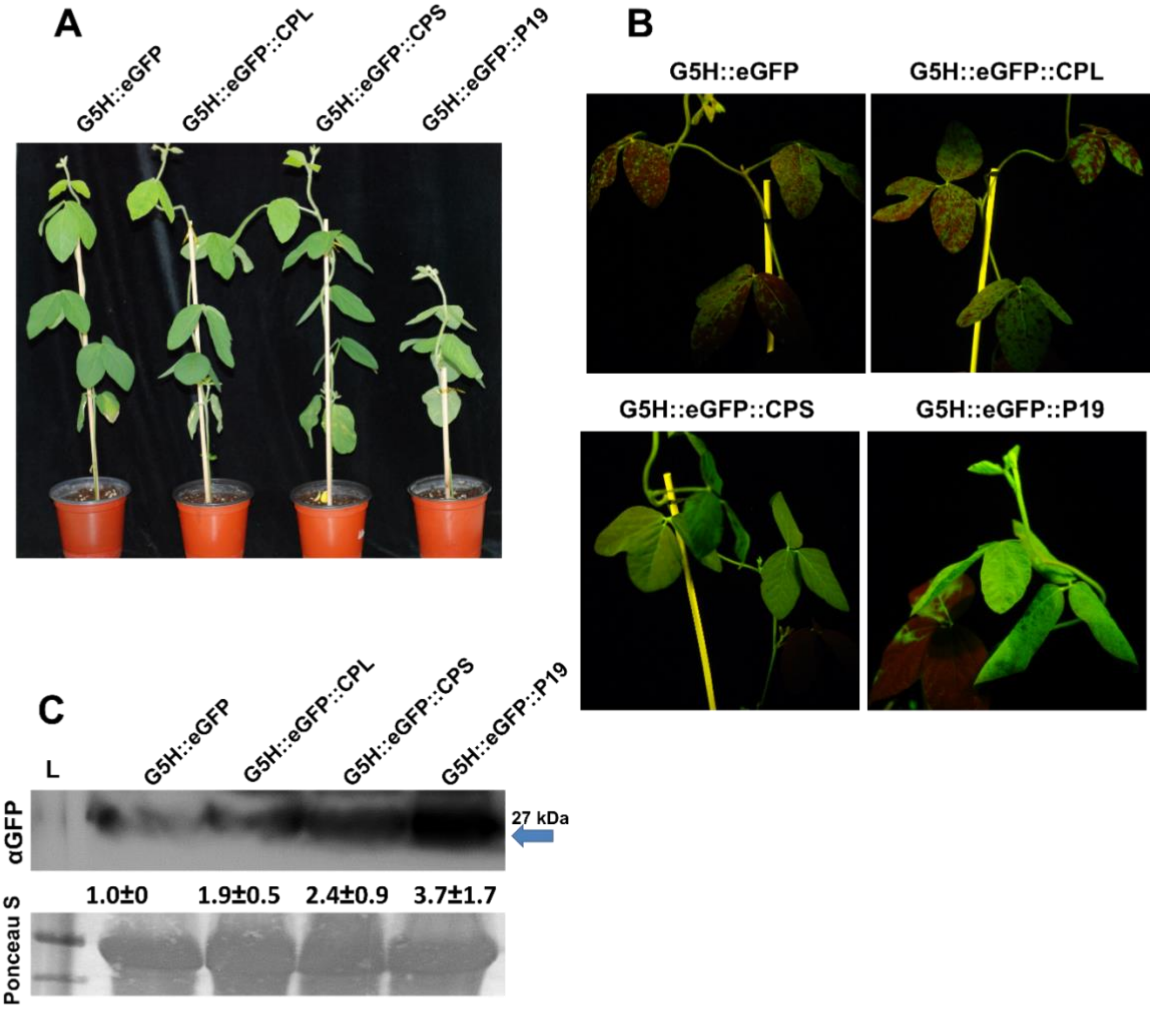

Supplemental Figure S3. Chimera constructions and the effect of CPL, CPS, or P19 on G5H strain of Soybean mosaic virus (SMV-G5H) virulence in Lee74 plants. A, Illustration of pSMV::eGFP backbone for both G5H::eGFP and G7H::eGFP. GUS gene replaced eGFP in the G5H::GUS infectious clone. Bean pod mottle virus (BPMV)- encoded VSRs CPL and CPS, as well as P19, were cloned into the SMV-G7H::eGFP and SMV-G5H::eGFP infectious clones downstream eGFP gene. B, Symptoms developed on Lee74 plants infected with G5H::eGFP expressing CPL, CPS, or P19. C, GFP fluorescence from Lee74 infected plants from (B). d Protein blot for eGFP from infected plants from (B). The numbers below the blot represent the

quantitative analysis of eGFP protein from three biological replicates. Ponceau S was used as a loading control.

Supplemental Figure S4

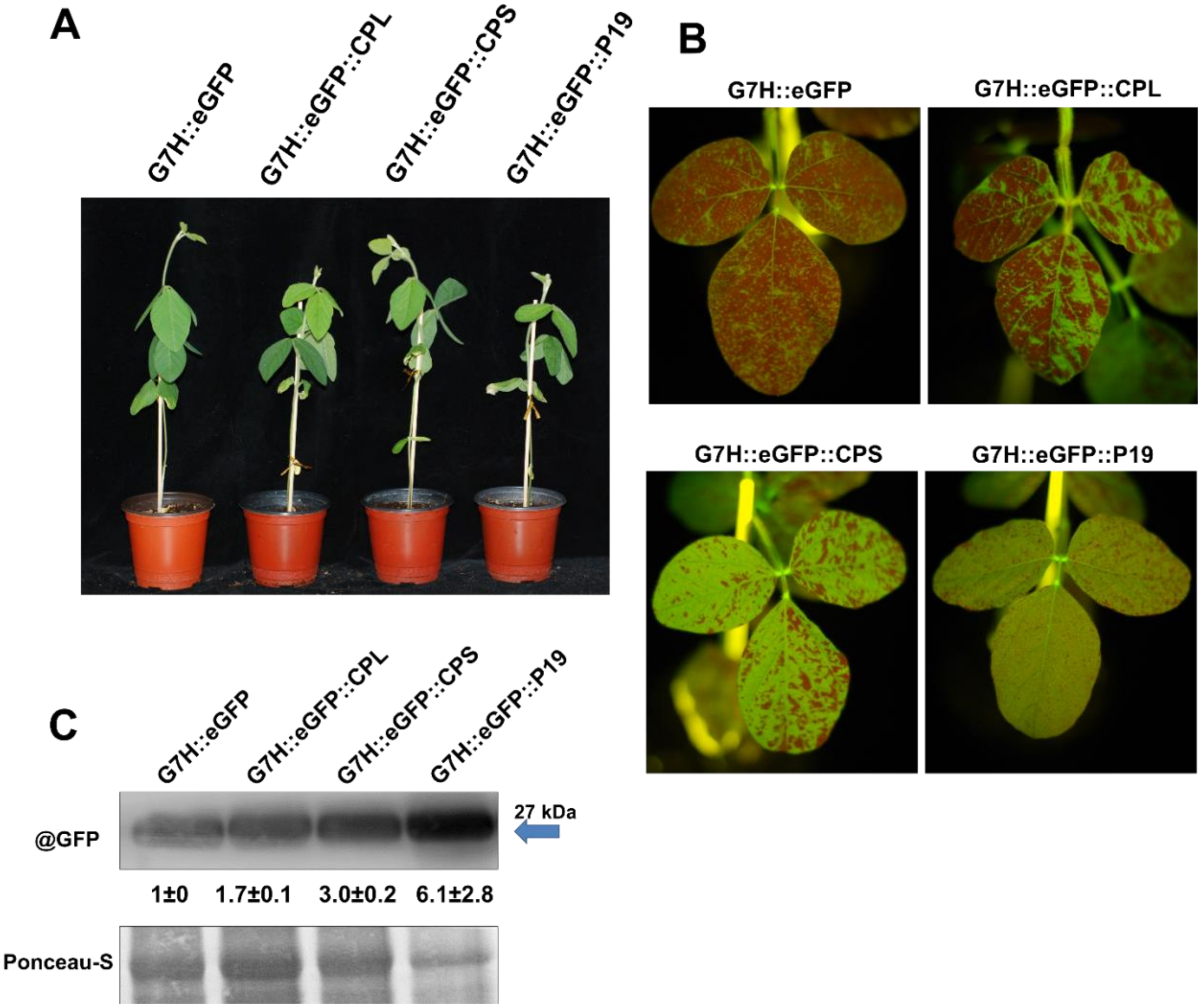

Supplemental Figure S4. Effect of the viral suppressors of RNA silencing (VSRs) CPL, CPS, and P19 on G7H virulence in Lee74 plants. Bean pod mottle virus (BPMV) encoded VSRs CPL and CPS, as well as P19, were cloned into the SMV-G7H::eGFP infectious clone downstream eGFP gene as described in Fig. S2. A, Symptoms developed on Lee74 plants infected with G7H::eGFP expressing CPL, CPS, or P19. B, GFP fluorescence from Lee74 infected plants from (A). C, Protein blot for eGFP from infected plants from (B). The numbers below the blot represent the quantitative analysis of eGFP protein from three biological replicates. Ponceau S was used as a loading control.

Supplemental Figure S5

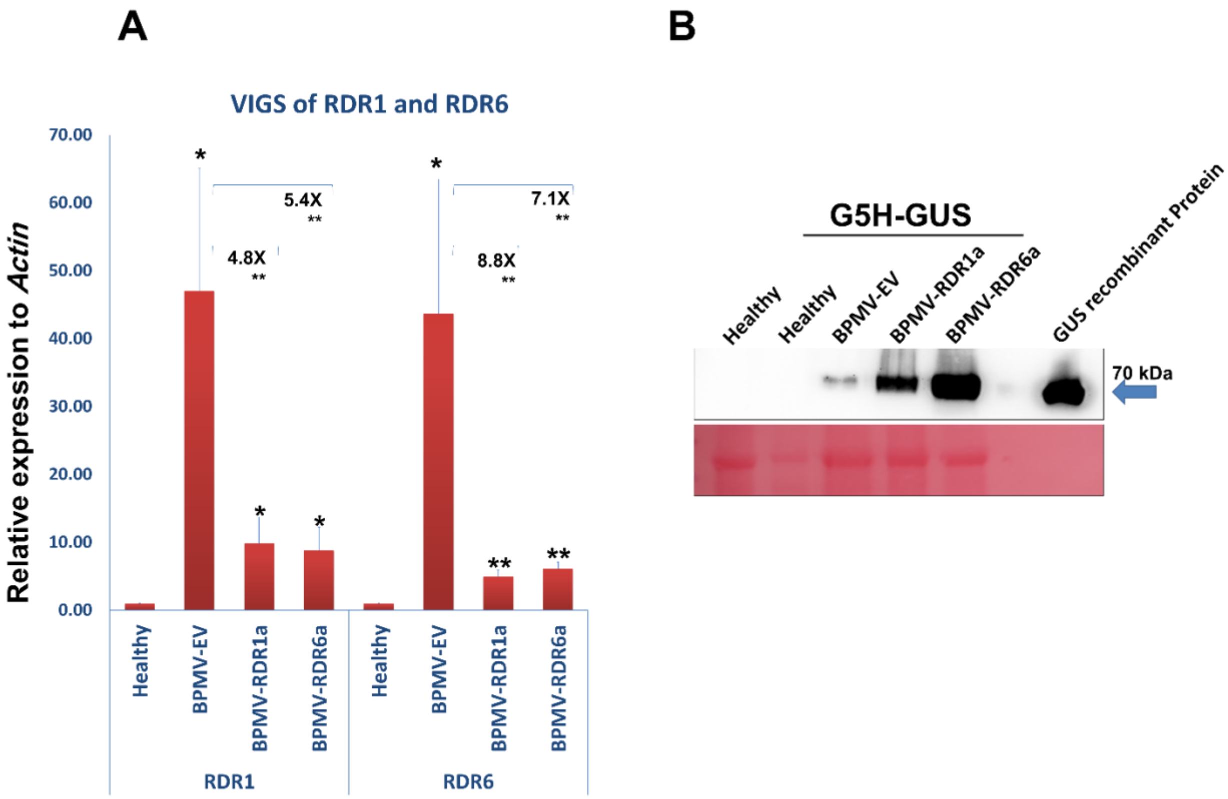

Supplemental Figure S5. Effect of silencing *RDR1a* and *RDR6a* on Rsv3-mediated resistance to SMV-G5H-GUS. A, Expression levels of *RDR1a* and *RDR6a* in silenced plants (Rsv3-plants) measured by qPCR. *Actin11* was used as an internal control. Values are means + SD of three biological replicates. A one-sided student t-test was used to determine the significant difference at $P < 0.05$ (*) and $P < 0.01$ (**). B. accumulation of SMV-G5H-GUS in Rsv3-silenced plants. B, Protein blots of SMV-G5H::GUS in the inoculated leaves at 5 dpi in healthy plants on plants pre-infected with Bean pod mottle virus (BPMV)- constructs. Ponceau-S was used as a loading control. Blot represents one of three biological replicates.

Supplemental Figure S6

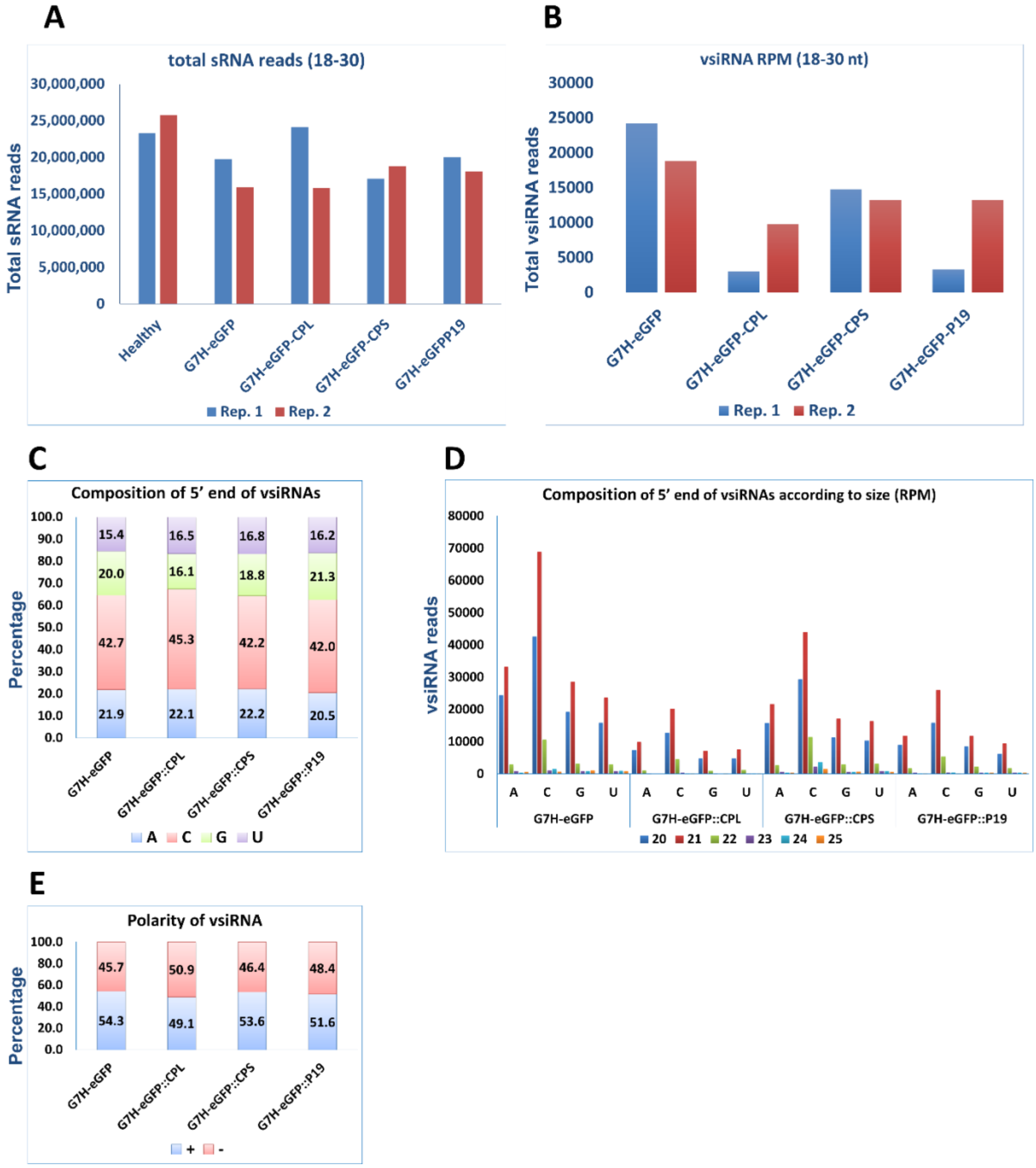

Supplemental Figure S6. Characteristics of sRNAs and vsiRNA in SMV-infected soybean. Characteristics of sRNAs and vsiRNA in SMV-infected soybean. A, total sRNA reads (18-30 nt) from two biological replicates. B, total vsiRNA reads per million (RPM) (18-30nt) in two biological replicates. C, the composition of the 5' end of vsiRNAs. D, Nucleotide composition of the 5′-end of the vsiRNA from L29 plants infected with SMV-G7H::eGFP, Soybean mosaic

virus (SMV) constructs; SMV-G7H::eGFP::CPL, SMV-G7H::eGFP::CPS, or SMV-G7H::eGFP19. E, the polarity of vsiRNAs.

Supplemental Figure S7

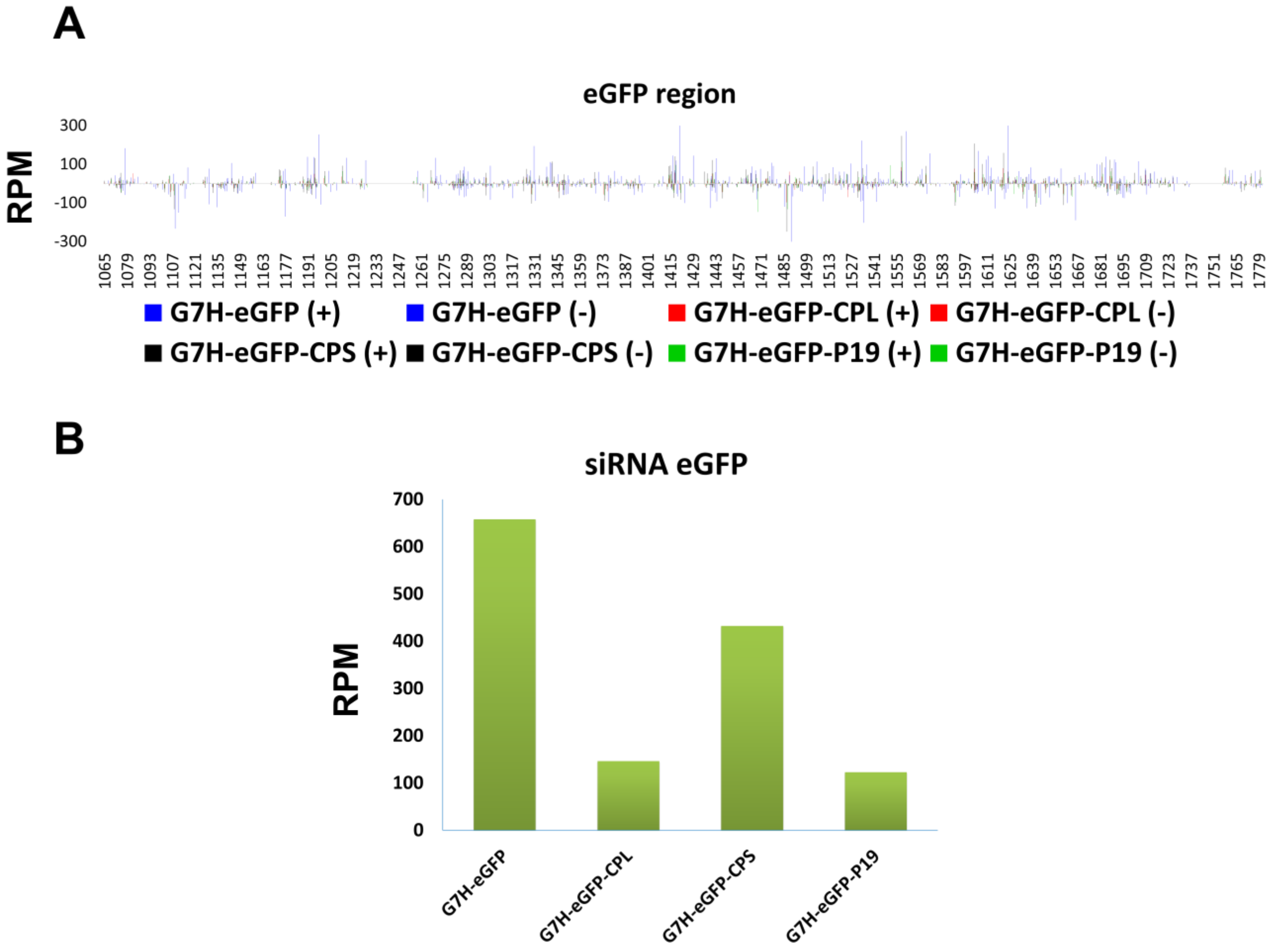

Supplemental Figure S7. siRNA eGFP distribution and levels. A Distribution of sen se (+) and antisense (-) eGFP-derived siRNAs along the G7H::VSRs::eGFP backbones expressing CPL, CPS, or P19 from L29 infected plants. B, Read per million of the si RNA-eGFP from (A). VSR: Viral suppressor or RNA silencing.

Supplemental Figure S8

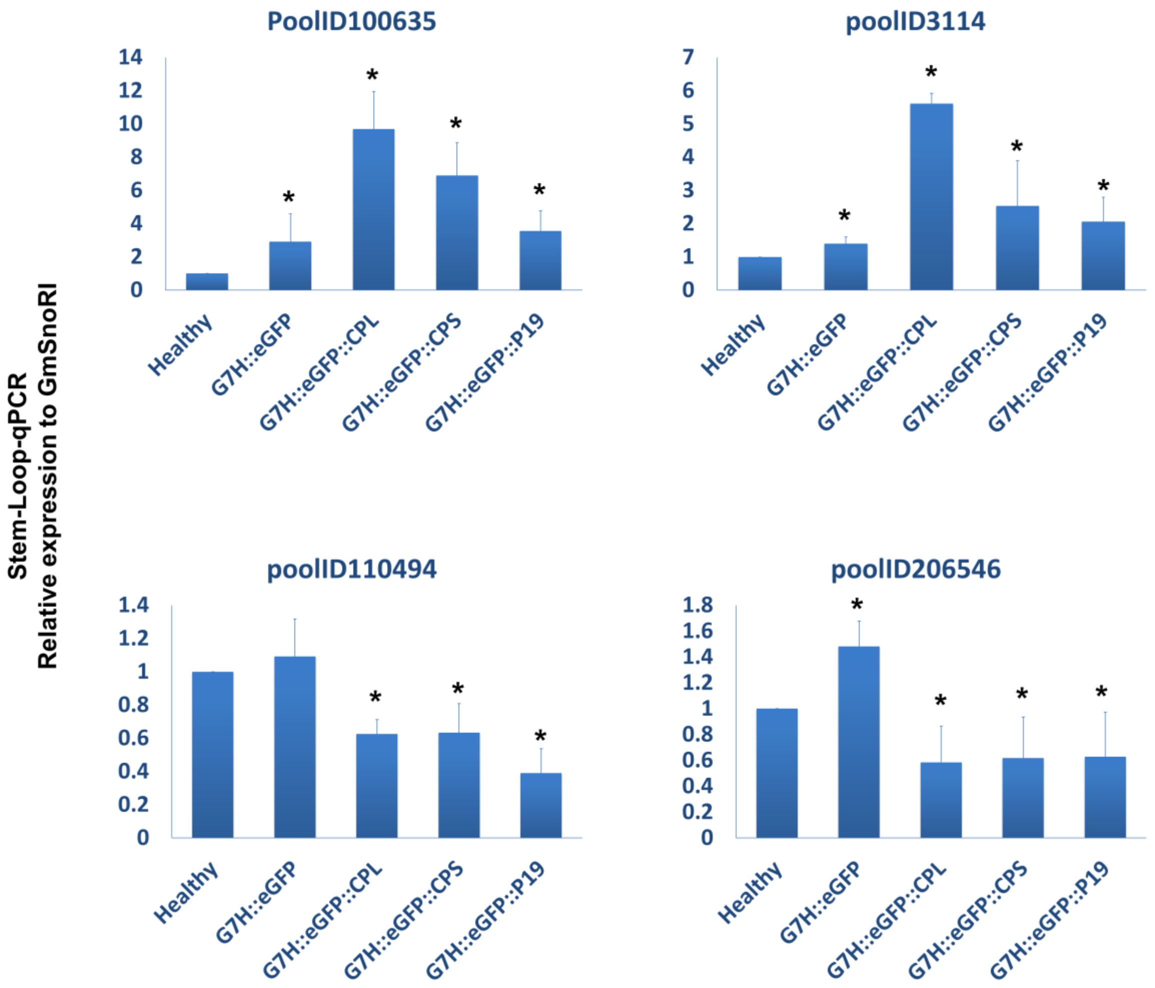

Supplemental Figure S8. Stem-loop qPCR for selected vasiRNAs from Fig. 6A. Two upregulated vasiRNAs (poolID3114 and poolID100635) and two downregulated vasiRNAs (poolID110494 and poolID206546) were selected from the list of vasiRNAs from Fig. 6A. GmSnoRI was used as an internal control. Values are means + SD of three biological replicates. A one-sided student t-test was used to determine the significant difference at $P < 0.05$ (*).

Supplemental Figure S9

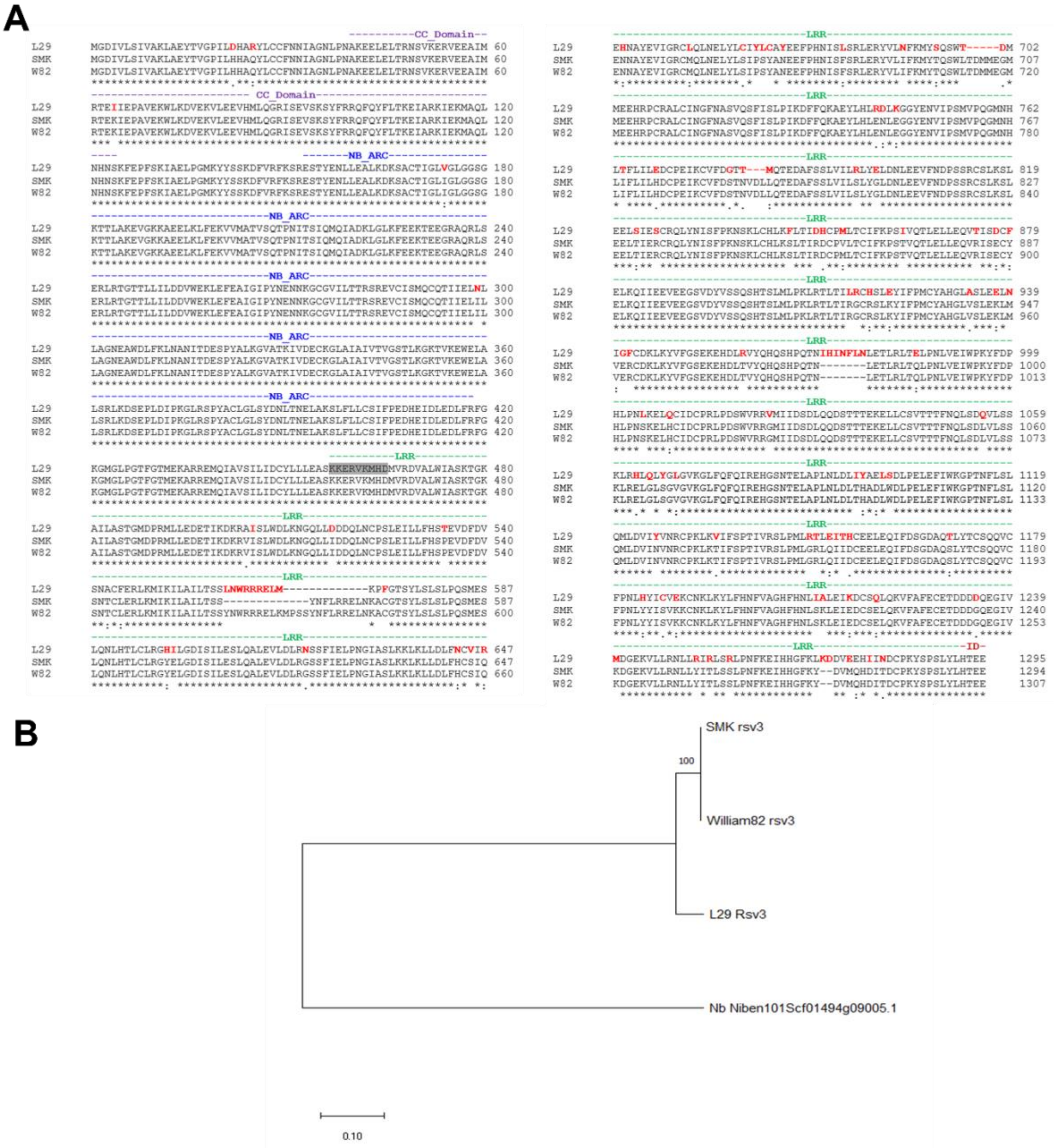

Supplemental Figure S9. Sequence alignment and phylogenetic analysis of Rsv3 orthologs. A, Coding sequence alignment between Rsv3 from L29 and rsv3 from William 82 (W82) and Somyongkong (SMK) soybean cultivars. The colored domains are Coiled Coil (CC) Domain, nucleotide-binding (NB) ARC- Domain, and leucine-rich repeat (LRR) domain. Red letters indicate amino acids absence or mismatch. B, Phylogenetic tree constructed based on

the amino acid (aa) sequences of Rsv3 from L29, rsv from W82 and SMK, and the *N. benthamiana* disease resistance nucleotide-binding leucine-rich-repeat receptors (NLR) protein (Niben101Scf01494g09005.1), which is the closest NLR to Rsv3 in the *N. benthamiana* database. A scale of distance is shown below.

Supplemental Figure S10

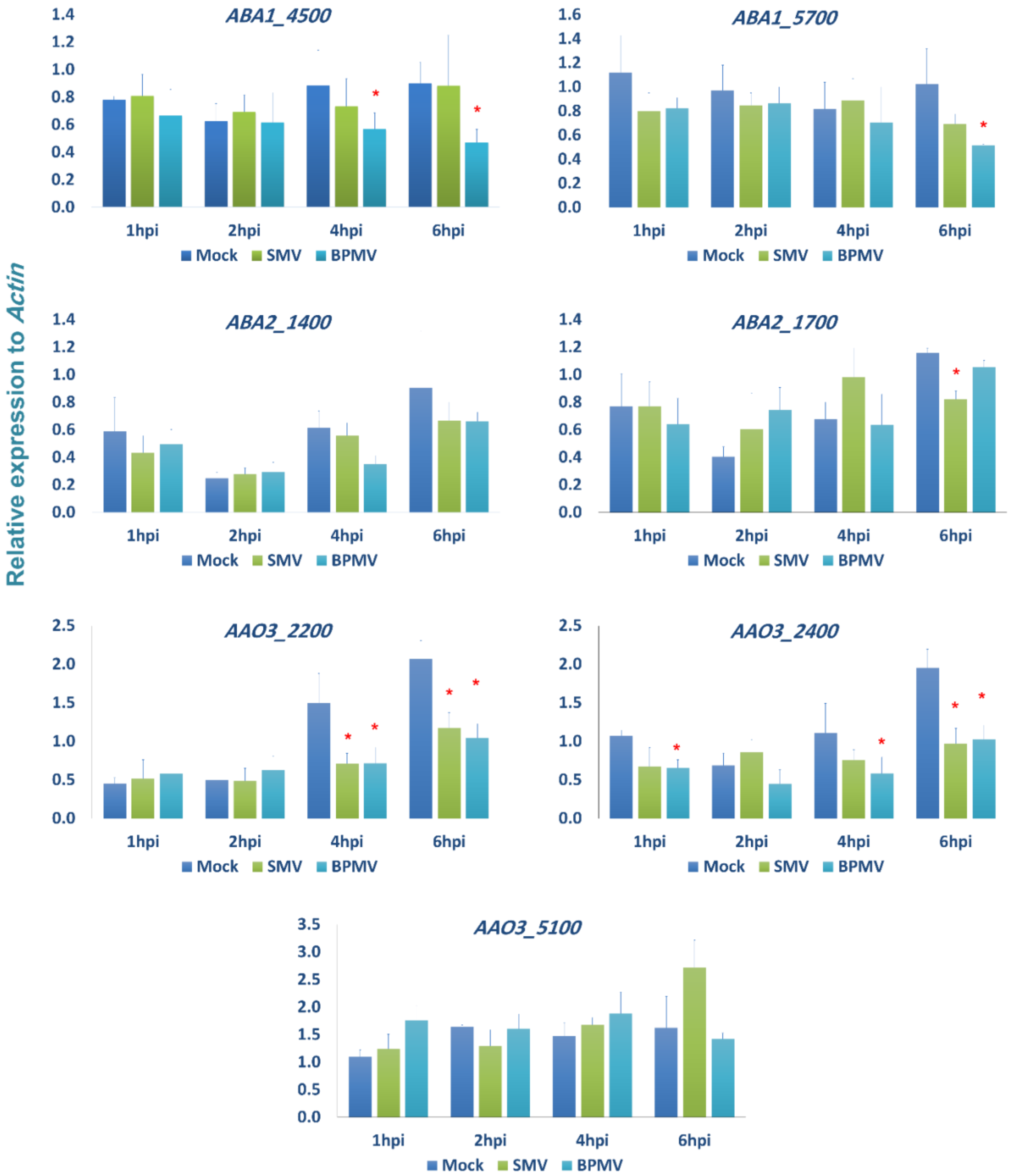

Supplemental Figure S10. Expression of ABA-related genes in L29 (Rsv3) plants infected with the G5H strain of soybean mosaic virus (SMV) SMV-G5H or with Bean pod mottle virus (BPMV). Values are means + SD of three biological replicates. A one-sided student t-test was used to determine the significant difference at P< 0.05 (*).

Supplemental Figure S11

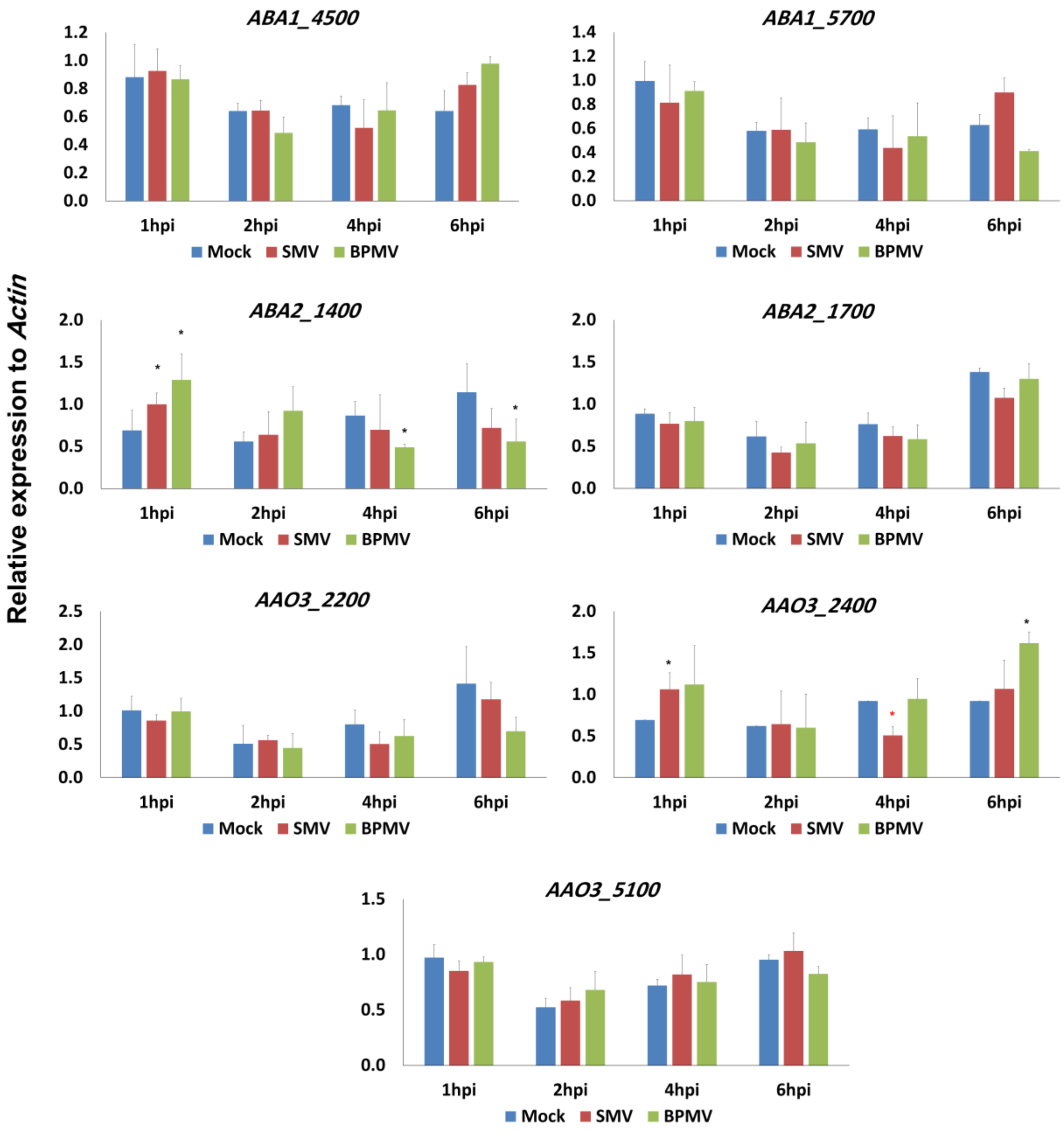

Supplemental Figure S11. Expression of ABA-related genes in SMK (rsv) plants infected with the G5H strain of soybean mosaic virus (SMV) SMV-G5H or with Bean pod mottle virus (BPMV). Values are means + SD of three biological replicates. A one-sided student t-test was used to determine the significant difference at P< 0.05 (*). Black and red asterisks indicate significant increase and decrease, respectively.
